# Can guidance during play enhance children’s learning and development in educational contexts? A systematic review and meta‐analysis

**DOI:** 10.1111/cdev.13730

**Published:** 2022-01-12

**Authors:** Kayleigh Skene, Christine M. O’Farrelly, Elizabeth M. Byrne, Natalie Kirby, Eloise C. Stevens, Paul G. Ramchandani

**Affiliations:** ^1^ Faculty of Education PEDAL Research Centre University of Cambridge Cambridge UK; ^2^ Present address: School of Education and Lifelong Learning University of East Anglia Norwich UK

## Abstract

This systematic review and meta‐analysis considered evidence of guided play compared to direct instruction or free play to support children's learning and development. Interventions from 39 studies were reviewed (published 1977–2020); 17 were included in meta‐analysis (*N*_total_ = 3893; *M*_childage_ = 1–8 years; *M*_girls_ 49.8%; *M*_ethnicity_ White 41%, African American/Black 28%, Hispanic 19%). Guided play had a greater positive effect than direct instruction on early maths skills (*g* = 0.24), shape knowledge (*g* = 0.63), and task switching (*g* = 0.40); and than free play on spatial vocabulary (*g* = 0.93). Differences were not identified for other key outcomes. Narrative synthesis highlighted heterogeneity in the conceptualization and implementation of guided play across studies.

**Abbreviations**
ECE: Early Childhood Education
EYFS: Early Years Foundation Stage
PRISMA: Preferred Reporting Items for Systematic Reviews and Meta‐Analyses
RCT: randomized controlled trial

There has been a longstanding debate in Early Childhood Education (ECE) concerning the relative benefits of free play and direct instruction for children's learning and development (Yu et al., 2018). In recent years there has been a conceptual shift toward a “play‐based learning” approach that acknowledges the combined benefits of play and traditional teaching, particularly in an ECE context (Fisher et al., 2011). Guided play is an educational approach that falls under the umbrella of “play‐based learning” (Hirsh‐Pasek et al., 2009; Zosh et al., 2018). In redefining play as a spectrum with varying degrees of child autonomy and adult guidance, guided play has been situated as a “middle‐ground” between free play and direct instruction. The intersection of play and guidance is believed to offer a powerful vehicle for early learning, harnessing the motivation and exploration that children benefit from during free play, and a Vygotskian‐inspired recognition that children's learning and development can be extended when effectively supported or “guided” by an experienced partner (Pyle & Danniels, 2016; Weisberg et al., 2016; Zosh et al., 2018).

Although it is generally accepted that play supports children's development and learning, until recently the field has lacked a clear evidence base regarding the benefits of playful learning (Whitebread, 2019). While several studies have found positive links between guidance during play and children's academic and socioemotional outcomes, including spatial language production, vocabulary development, and prosocial skills (Coplan et al., 2010; Ferrara et al., 2011; Han et al., 2010), it is difficult to draw robust conclusions about the overall benefit of guided play due to the diffuse nature of the literature. This is partly because the term “guided play” is not always ascribed to playful learning experiences that include its characteristics. Indeed, the concept has longstanding roots in several social‐developmental theories, which has given rise to inconsistencies in how playful learning conditions are described. Thus, there is a need to understand the similarities and differences in the ways that studies use guidance during play. To date, there has been no systematic synthesis of the evidence and questions remain about the effectiveness, conceptualization, and quality of guided play interventions and their evaluations. This evidence may have important implications for ECE practice, particularly for early years policy and curricula.

## Guided play and the theoretical basis for effectiveness

According to recent conceptualizations, guided play has three fundamental characteristics that combine to provide an optimal learning experience (Weisberg et al., 2013). First, the adult providing guidance should have a clear learning goal in mind when setting up a playful activity (Toub et al., 2018; Weisberg et al., 2016). Second, the activity or interaction should allow children some degree of choice and agency over their play: whether the playful interaction is adult‐ or child‐initiated, play should be child‐led where possible (Hirsh‐Pasek et al., 2009). Finally, the adult should be flexible in their use of guidance techniques (e.g., by using open‐ended questions, hints, prompts, modeling) to ensure sensitivity to the child's interests and needs. This requires the guiding adult to notice, interpret, and respond to a child's cues (Fisher et al., 2011). The combination of these features is believed to render guided play a particularly powerful context for learning, compared with free play or didactic learning alone. Specifically, in guided play, the learning experience is inherently meaningful to the child as play naturally cultivates their enjoyment, motivation, and agency; while the inclusion of guidance by a supportive adult extends the scope for learning beyond what the child might achieve on their own (Hopkins et al., 2019; Weisberg et al., 2016). It is important to note that the term guided play does not have to relate to a specific type of play, indeed it could include elements of several types including physical play, object play, and pretend and sociodramatic play.

While the label “guided play” is relatively recent, there is a strong conceptual basis grounded in developmental theory for its potential effectiveness. Assistance during play is widely believed to foster children's learning by providing them with more opportunities for active participation and self‐reflection than what is afforded by traditional didactic teaching methods (Smith, 1980; Sylva, 1984). Indeed, the interconnection between freedom and guidance, where the adult observes a child's play with interest to determine when and how to intervene to support their learning, is a central tenet of Froebelian philosophy of education (Bruce, 2015). An early form of guided play can be seen in “play tutoring” studies in 1970–1990s following on from Smilansky’s (1968) work which sought to enhance children's sociodramatic play through the provision of adult support and appropriate environments.

The value of active learning and adult support is highlighted in developmental theories which underpin many existing ECE practices, such as Vygotsky’s (1978) Zone of Proximal Development and scaffolding (Wood et al., 1976) and Rogoff's theory of guided participation (Rogoff, 2003). From the perspective of sociocultural theory, guided play aims to allow children to engage with and learn from their environment while receiving adult support that is contingent on their individual needs and interests (Weisberg et al., 2016). Guidance at appropriate times, for example, when a child appears to find an activity too difficult or too easy, can help them learn beyond what might be possible in independent play (Hannikainen & Munter, 2018; Van de Pol et al., 2012). Moreover, children's active engagement in a learning situation is believed to prepare them for future learning experiences (Rogoff, 2008). In this way, these engaged learning encounters may provide a fertile practice ground for skills such as confidence and critical thinking that children can take with them to future learning situations (Hopkins et al., 2019).

The use of guidance during play is considered especially valuable given that *play* may afford several benefits to children's learning. Various characteristics that are often present during play, such as positive emotion, meaningful contexts, active engagement, and social interaction, can have a facilitative effect on children's learning (Hirsh‐Pasek et al., 2015; Zosh et al., 2017). This aligns with Piagetian theory which suggests that play fosters learning as it allows information to be gathered in meaningful and intrinsically motivating ways (Piaget, 1972; Wood & Bennett, 1998). Similarly, self‐determination theory emphasizes that agency, experienced by children as they play, engenders their motivation to engage and learn (Deci & Ryan, 2000). Activities that are meaningful and enjoyable can support enhanced memory, attention, and motivation, all of which are important factors for learning (Bodrova et al., 2018; Bunzeck et al., 2012; Dang et al., 2012).

Given the potential of guided play as a privileged context for children's learning, a review of the literature is needed to evaluate the evidence, and to determine whether guided play is well‐suited to certain learning contexts and outcomes (Weisberg & Zosh, 2018).

## Guided play in policy and practice

Policy developments in the United States and the United Kingdom toward a greater academic focus have triggered debate about the role of play‐based learning in ECE settings (Martlew et al., 2011; Pyle & Danniels, 2016; Russell, 2011). The UK’s Early Years Foundation Stage (EYFS) Framework (Department for Education, 2018, 2020) and the USA’s “No Child Left Behind Act” (2002) both outline extensive academic targets which children are expected to achieve within their first few years of formal schooling (Hirsh‐Pasek et al., 2009). Curriculum pressures can seem incompatible with playful environments resulting in classrooms favoring direct instruction (Fisher et al., 2011; Hirsh‐Pasek et al., 2009; Pyle & Danniels, 2016). This bifurcation of play and learning in policy and curricula has been linked to ECE teachers’ reports that they feel uncertain about how to deliver learning through play (Bubikova‐Moan et al., 2019).

An understanding of the state of the evidence regarding the effectiveness of play‐based learning is needed to inform decisions about teacher education and professional development (Bubikova‐Moan et al., 2019; Pyle & Danniels, 2016). Clarity is needed regarding how play‐based learning might be effectively implemented in a way that is manageable for teachers (Pramling et al., 2019).

## Existing reviews

This is the first review to examine the effectiveness of guided play interventions on children's learning. There are several existing reviews which consider one of the components of guided play; specifically, the impact of adult guidance during children's learning outside of play. A meta‐analysis of 360 studies that compared guidance during discovery‐based learning (in which children learn through independent exploration) to traditional teaching, found a small effect (*d* = .30) on learning outcomes (Alfieri et al., 2011). Positive effects were domain‐specific with large effects observed for computer‐based skills, medium effects for verbal and social skills, and smaller effects for maths and science outcomes. A review of 72 studies examining maths and science outcomes revealed a positive effect of guidance (*d* = .50) versus no‐guidance during inquiry‐based learning, where children learn through self‐directed investigations (Lazonder & Harmsen, 2016). Existing reviews have suggested that lower levels of guidance may increase levels of cognitive demand on children by exceeding the limitations of their working memory capacity. This suggests that there may be optimal levels of guidance for learning to occur (Kirschner et al., 2006; Lazonder & Harmsen, 2016; Mayer, 2004). However, the optimal level of adult guidance may depend on the target outcome (Hobbs et al., 2019). Therefore, there is a need to consider how, and to what degree, adult guidance is implemented during interventions to better understand its potential effectiveness for learning and development (Fisher et al., 2011; Yu et al., 2018).

Crucially, the existing reviews do not focus on play contexts, thus the additional affordances that play contexts may offer, particularly for young children, have not yet been subject to systematic review. One notable exception is a review of six studies involving the “Tools of the Mind” play‐based curriculum. The intervention had a small but significant positive effect on maths (*d* = .06) but not literacy or self‐regulation outcomes (Baron et al., 2017). Tools of the Mind has some features which may overlap with guided play. For example, teachers support children to produce learning‐focused “play plans” which they use to act out their own play scenarios (so the guidance is occurring before, rather than during the play (Barnett et al., 2008). However, the effects of this guidance cannot be disentangled from the impact of other activities in the program.

## Rationale and objectives

Developmental theories suggest that combining adult guidance and child agency in educational contexts could benefit a wide range of learning and developmental outcomes (Vygotsky, 1978). While existing research suggests that guided play is a promising approach for promoting children's learning, the evidence is diffuse and results are mixed, particularly when guided play is contrasted with alternative classroom experiences like free play and direct instruction. Weisberg et al. (2013) and Zosh et al.’s (2018) recent conceptualizations of guided play provide a cohesive framework within which to synthesize the literature. Furthermore, due to the recent resurgence of play research (Whitebread, 2019), which includes important studies on guided play, this is an opportune moment to review the literature.

The current review aimed to collate and synthesize research studies that have investigated the effects of guidance during play on children's outcomes. Two key research questions were addressed: (1) *how effective are guided play interventions for improving children's learning and developmental outcomes compared to free play and direct instruction*, and (2) *how is guided play conceptualized and implemented within experimental studies with respect to adult guidance and child choice?* To address the first (primary) research question, results from multiple studies were combined for meta‐analyses (where appropriate) to determine an overall effect of guided play on various child outcomes, relative to free play or direct instruction. To address the secondary research question, a narrative synthesis approach was used to examine textual references to how guided play was both conceptualized and implemented across studies, and how these two concepts relate to each other. The research questions and analyses were pre‐specified, but no directional hypotheses were made.

## METHOD

### Protocol, registration, and reporting standards

A protocol was submitted to the PROSPERO registry for systematic reviews (CRD number: 42019153366). PRISMA guidelines of reporting were followed.

### Eligibility criteria

#### Types of studies

This review included randomized controlled trials (RCTs), in which participants were assigned randomly to either an intervention or control group; and non‐randomized (or quasi‐experimental) controlled trials, in which assignment was non‐random but where the study otherwise resembles a randomized field experiment (Remler & van Ryzin, 2010). Non‐randomized designs included counterbalance methods (in which the child is exposed to both conditions) where the study presented a distinction in results after each stage of counterbalancing. The searches were conducted with no restrictions based on publication date, language, or type of report (e.g., published journal article, thesis, conference report).

#### Types of participants

The population of interest was children with a mean age of 1–8 years (12–96 months), regardless of gender, ethnicity, developmental ability, or socio‐economic status. This age range was chosen as it is commonly used to define early childhood (World Health Organisation, 2020), the scope for child choice over their actions is limited for children under the age of 1 year, and studies involving children over the age of 8 years do not typically involve play or playfulness. Only studies in which a teacher, parent, or member of a research team provided guidance to the child were included (e.g., studies involving peer support or guidance from a computer were not included).

#### Types of settings

While the educational nature of guided play suggests that most studies would be carried out in ECE classrooms, studies were also included if they were carried out in laboratory‐based, museum, or home environments.

#### Types of interventions

Studies were included if they compared a curriculum, intervention, or activity involving guided play, to one or more control groups. Guided play was defined as an approach that involves: (a) child autonomy (a child has some freedom and choice over their own actions and play behavior), (b) adult guidance (an adult initiates the play experience and provides guidance using one or more of the following strategies: providing sensitive hints/prompts, asking open‐ended questions, setting challenges, guiding a child's attention by modeling, joining in the play [co‐play], and/or adapting to the individual needs, interests, and understanding of the child [scaffolding]), and (c) a learning goal (the play‐based task has a clear learning goal which the adult keeps in mind and guides the child toward). This definition is based on Weisberg et al.’s (2013) conceptualization of guided play and was further developed through consultation with an expert panel (see Appendix S1).

Eligible control conditions were free play or direct instruction/business‐as‐usual. Free play was defined as play which is initiated and directed by a child with no specified learning goal or adult involvement (Zosh et al., 2018), although the available materials may be constrained (e.g., blocks). Direct instruction was defined as the use of didactic teaching methods to explicitly instruct children of a skill or learning goal, during which the child has limited choice in the interaction (Weisberg et al., 2016). Only studies in which an intervention or exposure had been introduced by the research group were eligible for inclusion, meaning that studies in which natural exposure occurred were not included.

#### Types of outcome measures

For inclusion, studies had to assess at least one outcome relating to child learning and development in one of the following categories: cognitive and academic learning (e.g., language/literacy, maths/numeracy, science, or executive function/self‐regulation), socioemotional development (e.g., prosocial behavior, anxiety, behavior), or physical development (e.g., gross motor skills). The review aimed to map the outcomes that have been used in the existing guided play literature. A broad range of learning and development domains were considered for inclusion and no restrictions were placed on the outcome measures used. This reflects the broad range of outcomes that are likely to be relevant to ECE practitioners.

### Search methods for identification of studies

#### Information sources

Eight databases were selected following consultation with an information specialist. These included a broad range of relevant psychology and education journals that are also commonly searched in other reviews of education research. The databases were ERIC (EBSCO), BEI (EBSCO), Child Development and Adolescent Studies (EBSCO), PsycINFO (EBSCO), PsycARTICLES (EBSCO), Scopus, Web of Science, and OpenGrey (www.opengrey.eu/).

#### Search

An initial scope of the literature and consultation with an expert panel identified the following keywords: guided play, scaffolded play, enhanced play, facilitated play, assisted play, supported play, learning through play, play‐based learning, and purposeful play. Appropriate search options—including Boolean operators, MESH terms, and truncation operators—were used to construct and combine searches for each of the databases. Initial searches were conducted on October 17, 2019, with a supplementary search conducted on February 16, 2021. The full search strategy is provided in Supporting Information (see Tables S1 and S5). All electronic databases were set to email K.S. for a period of 8 months following the initial search to identify new articles which matched the search strategy. At regular intervals, these studies were collated and screened for relevance and included if appropriate. Articles were also identified through hand searching the reference lists of key papers and those that were screened as eligible for inclusion. A list of all studies identified for inclusion was shared with an expert panel to ensure key papers had not been missed. Any additional papers suggested were screened for inclusion.

### Data collection and analysis

Four authors were involved in screening papers for eligibility and in data extraction (K.S., E.M.B., C.O.F., and N.K.).

#### Study selection

All papers generated from the search strategy were exported to EPPI‐Reviewer 4 (Thomas et al., 2020). Two authors independently screened titles and abstracts of all articles for inclusion using the eligibility criteria. Another author independently screened the abstracts and titles of 10% of identified studies for reliability purposes. Disagreements between individual judgments occurred for 3% of papers and were resolved through discussion. Studies identified as eligible or “in need of further information” were then screened as full texts, again with 10% screened by an additional reviewer.

#### Data extraction and management

Two pairs of researchers independently extracted data from all studies that were included following full‐text screening. Discrepancies were discussed and resolved with a third reviewer when required.

A detailed code set, informed by the Cochrane Checklist (Higgins & Green, 2011), was used for data extraction; see Appendix S2 for a copy of the coding form. Data were extracted based on the inclusion and exclusion criteria, characteristics of the study setting and population (including the ethnicity and gender/sex make‐up of the sample), research design, and type of outcome measures. Further details about the intervention itself were also extracted (e.g., adult involved, setting, type of adult guidance used). To support narrative synthesis, information relating to the conceptualization and implementation of guided play was also extracted from the background and method sections of included studies. Where available, raw means and *SD*s of post‐test scores (or gain scores for those that only reported pre‐to‐post differences) were extracted. EPPI‐Reviewer 4 (Thomas et al., 2020) was used to calculate Hedges’ *g* effect sizes and 95% CIs. Where data were missing or unclear, authors were contacted to request further information. If numerical data were missing and authors could not provide this information, studies were included in the review but not in the meta‐analyses. Details of excluded studies are noted in Table S2.

#### Risk of bias in included studies

The quality of included papers was assessed using the Cochrane Risk of Bias Tool (Higgins & Green, 2011). The tool provides a consistent framework with which to assess risk of bias across the included papers—that is, the risk of over‐ or under‐estimating true intervention effects (i.e., internal validity). Each study was rated as having a “high,” “low,” or “unclear” risk of bias for the following domains: random sequence allocation, allocation concealment, blinding of participants and personnel, blinding of outcome assessment, handling of incomplete outcome data, selective reporting of outcomes, and other biases. The latter comprised pre‐specified confounds typical in education‐based research (e.g., the inclusion of a single teacher/class/school in the study and conflicts of interests—such as authors designing and delivering the intervention). Each eligible study was independently assessed by two authors (E.M.B. and K.S., or E.M.B. and N.K.) and any discrepancies were resolved through discussion with a third reviewer (C.O.F. or P.G.R.).

While the tool provides a standardized and transparent procedure for assessing the internal validity of trials, it is not without limitations. The validity of the tool is not yet well‐established, and modest levels of inter‐rater agreement have been reported—indicating subjectivity in making judgments of bias (potentially due to lack of clear guidelines; Jørgensen et al., 2016). Furthermore, items are often deemed “unclear” due to difficultly retrieving information that is not reported by authors, meaning a judgment regarding bias is not always possible, or that information is gathered from less reliable sources (i.e., not the manuscript; Faggion, 2016). Due to the dichotomous nature of the tool, and difficulty in judging bias of some items due to insufficient information being provided in the manuscripts, the tool may be less sensitive to between‐study heterogeneity. Nevertheless, the tool is widely used to evaluate the quality of trials and provides useful guidelines for standardizing the decision‐making process (Deeks et al., 2020).

#### Measures of treatment effects

For continuous outcome measures, the Hedges’ *g* statistic was used to measure the effect size between the post‐test scores of the intervention and comparison groups. For studies that compared guided play to both free play and direct instruction, separate effect sizes were calculated for each comparison condition. Meta‐analyses were only conducted for outcomes which had data from at least two studies using similar outcome measures.

#### Assessment of heterogeneity

The *I* ^2^ statistic quantifies heterogeneity by describing the degree of variance across studies (reported as a percentage) and is less dependent on the number of studies included in an analysis than other measures (Higgins et al., 2003). A higher *I* ^2^ value indicates greater heterogeneity.

#### Assessment of reporting biases

The effect size associated with the primary outcome measure for each intervention was plotted against the standard error in a funnel plot (created in EPPI‐Reviewer 4; Thomas et al., 2020). Egger's regression asymmetry test was then used to assess publication bias (Egger et al., 1997).

### Quantitative and qualitative synthesis

Following data extraction, findings were examined quantitatively and qualitatively. First, we conducted meta‐analyses of child outcomes and moderators of effects (more details on how these studies were selected are provided in subsequent sections). Then, all included studies were summarized using a narrative synthesis approach.

#### Meta‐analyses of child outcomes

Meta‐analyses combine the results of two or more studies to improve the precision of the estimates of effect (Deeks et al., 2020). In this review, studies with comparable outcomes were entered into meta‐analytic random effects models (accounting for within‐study variability), and results are reported according to outcome domains. All pooled effect size estimates along with 95% confidence intervals are presented as forest plots in Supporting Information. For outcome data that could not be included in a meta‐analysis, effect sizes for single studies were calculated and are reported. If numerical data were insufficient, and additional information could not be acquired from study authors, the main study findings are summarized descriptively.

#### Moderator analyses

Independent analyses were conducted for each of the following moderator variables: intensity of intervention exposure (1 to 5, 6 to 20, or >20 exposures), adult involved (parent, teacher, or researcher), number of child participants (<50 or ≥50), type of comparator (free play or direct instruction), and study design (randomized‐ or quasi‐experimental). Other pre‐specified analyses were not possible due to limited variation between studies (child age [1–3 years‐old, 4–6 years‐old, 7–8 years old] and country income inequality). Only studies that provided sufficient data (means and *SD*s) were included in these analyses. Eligible studies were pooled for moderator analyses (random effects meta‐analytic models), regardless of outcome measure; results are presented in forest plots in Supporting Information. Where individual studies assessed more than one outcome measure, a primary measure was identified for each study for inclusion in the moderator analysis.

#### Narrative synthesis approach

Narrative synthesis was used to examine textual references within studies to map the similarities and differences across studies in terms of their conceptualization and implementation of guided play (Popay et al., 2006). This approach generates findings based on words and text in a study, which in turn allows for the synthesis of heterogeneous interventions, as expected in the current review. Content analysis was used to investigate the theoretical conceptualization and implementation of the interventions at a deductive level based on two categories: the degree of child choice and flexible adult guidance included. A coding scheme was developed to guide the narrative synthesis (see Appendix S3). Three authors (K.S., C.O.F., and N.K.) extracted and coded data relating to concept and implementation of guided play. A fourth author (E.M.B.) coded 15% of studies and all discrepancies were discussed and resolved. As two dimensions were measured for each study (conceptualization and implementation), a quadrant of studies was created. All studies were included in the narrative synthesis.

## RESULTS

### Study selection

**FIGURE 1:**
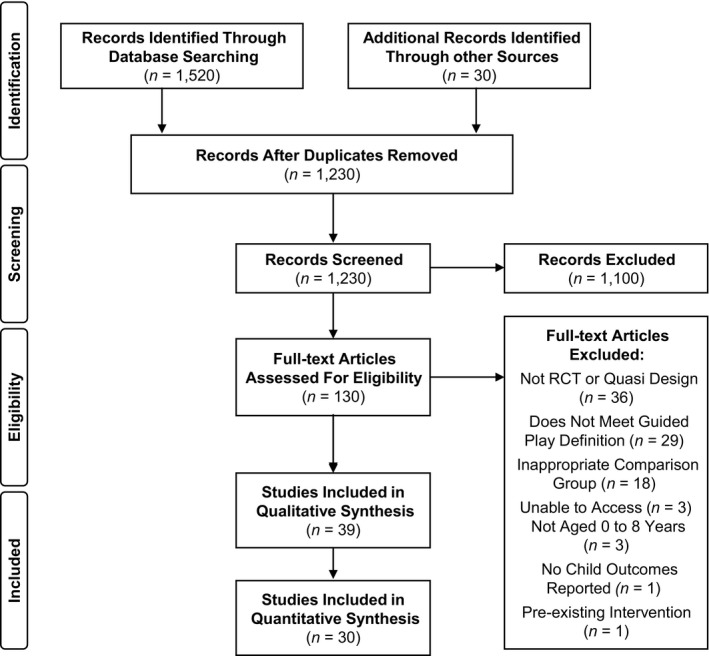
PRISMA flowchart of search results and included and excluded studies. The initial search was conducted on October 17, 2019 based on the search terms in the protocol. A supplementary search of electronic databases was conducted on February 16, 2021 based on additional search terms suggested during peer review. Of the 1230 studies included for screening, 138 were identified in the additional search, six of which were ultimately included in the final review

The systematic search results are displayed in a PRISMA flow diagram (Figure 1). In total, the search yielded 1230 potentially eligible studies following the removal of duplicates. 1200 studies were identified via electronic search and 30 through hand searching, searches of grey literature, and recommendations from an expert panel. Following eligibility screening, 130 studies were identified for full‐text screening. A total of 39 studies (from 38 papers) met the inclusion criteria and were included in the review. Reasons for exclusion are noted in the PRISMA flow diagram (Figure 1) and Table S2. Of the 39 studies, 17 were suitable for inclusion in a meta‐analysis of child outcomes as they shared at least one common outcome measure with another study. Thirty studies also provided sufficient data (means, *SD*s) for inclusion in moderator analyses.

### Study characteristics

The search identified 22 RCTs and 17 quasi‐experimental studies. Of these, 23 compared guided play to direct instruction, nine compared guided play to free play, and seven included both as comparison groups. See Table 1 for summary of study characteristics. Thirty‐four studies were conducted in countries with medium‐income inequality (*n* = 24 in the United States, one each in Switzerland, Turkey, Portugal, China, and Canada, two in the United Kingdom, and three in Australia). Of the remaining five studies, four were conducted in countries with low‐income inequality (Belgium, Denmark, the Slovak Republic, and the Netherlands) and one was conducted in countries with both medium‐ and high‐income inequality (Kenya and South Africa, respectively). Studies varied according to participant characteristics, intervention delivery, outcomes measured, and guided play content, which are considered in turn below.

**TABLE 1:** Characteristics of included studies (*N* = 39)

Author (year)	Study design	Child's age (years)	Child development	Intervention location	Adult present	Country	Income inequality	Total participants	Number of exposures	Main outcome(s)	Control type
Bierman (2015)	RE	3–6	Typical	Home	Parent	USA	Medium	100–199	6–20	Literacy and social skills	DI
Bleses (2020)	RE	1–3	Typical	ECC	Teacher	Denmark	Low	200+	21+	Language and maths	DI
Borriello (2018)	QE	3–6	Typical	Laboratory	Parent	USA	Medium	0–49	1–5^b^	Maths/spatial language	FP
Bulunuz (2013)	QE	3–6	Typical	Classroom	Teacher	Turkey	Medium	0–49	21+	Science concepts	DI
Casey (2008)	QE	3–6, 6–8	Typical	Classroom	Teacher	USA	Medium	100–199	6–20	Maths‐spatial skills	DI
Cavanaugh (2017)	QE	3–6	Typical	Classroom	Teacher	USA	Medium	0–49	6–20	Literacy/language	DI
Christie (1983)	QE	3–6	Typical	Classroom	Researcher	USA	Medium	0–49	6–20	Literacy—vocabulary, play, and creativity	DI
Cohrssen (2019)	QE	3–6	Typical	Classroom	Teacher	Australia	Medium	50–99	21+	Maths skills	DI
Conner (2013)	QE	1–3	Typical	ECC	Researcher	USA	Medium	0–49	6–20	Literacy/language	FP
Coplan et al. (2010)	RE	3–6	Typical	ECC	Researcher^a^	Canada	Medium	0–49	6–20	Social skills	DI
Dejonckheere (2016)	RE	3–6	Typical	Classroom	Teacher	Belgium	Low	50–99	Unclear	Exploratory play	DI
Dempsey (2013)	RE	1–3, 3–6	Typical, additional needs	Home, ECC	Parent	USA	Medium	0–49	21+	Play (pretend and exploratory)	FP
Dickinson (2019)	RE	3–6	Typical	Classroom	Teacher	USA	Medium	200+	21+	Literacy—vocabulary	DI
Eason (2020)	RE	3–6	Typical	Laboratory	Parent	USA	Medium	50–99	1–5^b^	Maths vocabulary	DI, FP
Ferrara et al. (2011)	RE	3–6	Typical	Unclear	Parent	USA	Medium	0–49	1–5^b^	Maths—spatial language	FP
Fisher (2011a)	RE	3–6	Typical	Laboratory	Researcher	USA	Medium	0–49	1–5^b^	Maths—shape knowledge	DI
Fisher (2011b)	RE	3–6	Typical	Classroom	Researcher	USA	Medium	0–49	1–5^b^	Maths—shape knowledge	DI, FP
Fisher (2013)	QE	3–6	Typical	Classroom, Laboratory	Researcher	USA	Medium	50–99	1–5^b^	Maths—shape knowledge	DI, FP
Gmitrova (2013)	RE	3–6	Typical	Classroom	Teacher	Slovak Republic	Low	200+	1–5^b^	Play	FP
Goldstein (2018)	RE	3–6	Typical	Classroom	Researcher	USA	Medium	50–99	21+	Emotional control	DI
Golomb (1977)	QE	3–6	Typical	Unclear	Researcher	USA	Medium	0–49	1–5	Conservation judgment	DI
Han et al. (2010)	RE	3–6	Typical	Classroom	Researcher^a^	USA	Medium	0–49	21+	Literacy—vocabulary	DI
Jemutai (2019)	QE	3–6, 6–8	Typical	Classroom	Teacher	South Africa, Kenya	High, Medium	50–99	21+	Visuospatial abilities	DI
Kalkusch (2020)	RE	3–6	Typical	ECC	Researcher	Switzerland	Medium	50–99	6–20	Pretend play	FP
Lau (2005)	QE	3–6	Typical, additional needs	Classroom	Teacher	USA	Medium	0–49	21+	Social interactions	FP
Li (2016)	RE	3–6	Typical	Classroom	Unclear	China	Medium	0–49	6–20	Social skills	DI
Morris (2018)	RE	3–6	Typical	Classroom	Teacher	Australia	Medium	200+	6–20	Knowledge of wellbeing/sustainability	DI
O’Connor (2011)	QE	3–6, 6–8	Additional needs	Classroom	Therapist	Australia	Medium	0–49	21+	Play, language and social skills	DI
Palma (2014)	RE	3–6	Typical	Classroom	Teacher	Portugal	Medium	50–99	21+	Gross motor skills	DI, FP
Pearson (2008)	RE	3–6	Typical	Classroom	Researcher	USA	Medium	0–49	1–5	Problem solving and emotions	FP
Pellegrini (1980)	QE	3–6	Typical	ECC	Researcher	USA	Medium	0–49	1–5^b^	Language‐associative fluency	DI, FP
Sawyer (2019)	RE	3–6	Typical	Classroom	Researcher	USA	Medium	50–99	21+	Drawing development	DI
Schmitt (2018)	RE	3–6	Typical	Classroom	Researcher	USA	Medium	50–99	6–20	Maths and executive function	DI
Sinha (2012)	QE	3–6	Typical	Classroom	Researcher	USA	Medium	50–99	1–5	Self‐regulation	DI, FP
Smith (1978)	QE	3–6	Typical	Private residence	Unclear	UK	Medium	0–49	6–20	Play and social participation	DI
Smith (1981)	QE	3–6	Typical	ECC	Researcher^a^	UK	Medium	50–99	21+	Social participation, cognitive ability (literacy/language, maths, visuospatial) and play	DI
Thibodeau (2016)	RE	3–6	Typical	Classroom	Researcher	USA	Medium	50–99	21+	Executive function	DI
Toub et al. (2018)	RE	3–6	Typical	Classroom	Researcher	USA	Medium	200+	6–20	Literacy—vocabulary	DI, FP
van schijndel (2010)	QE	1–3	Typical	ECC	Teacher	Netherlands	Low	0–49	6–20	Exploratory play	FP

Abbreviations: DI, direct instruction/treatment as usual; ECC, Early childhood care and education setting; FP, free play; QE, quasi‐experimental design; RE, randomized experimental design.

^a^ Outside member, e.g., tutor, group leader, but trained by research team.

^b^ Single exposure to intervention.

#### Participant characteristics

Approximately half the studies (*n* = 19) had <50 participants, 13 included 50–199 participants, and only five (all RCTs) included ≥200 participants. In total, the studies involved 3893 child participants, but participant numbers varied considerably across studies, with sample sizes ranging from nine (Dempsey, 2013) to 1116 (Bleses, 2020). Variation in age was limited: most studies (*n* = 32) exclusively reported on participants aged 3–6 years. Four studies included children aged 1–3 (Dempsey, 2013) or 6–8 years (Casey, 2008; Jemutai, 2019; O’Connor, 2011), and another three included participants aged 1–3 years. Most studies (*n* = 36) reported on typically developing children only, while O’Connor (2011) exclusively included children with intellectual disabilities, and Lau (2005) and Dempsey (2013) reported on both typically developing children and those with additional needs. Coplan et al. (2010) and Li (2016) reported on children who were rated by parents as being “extremely shy,” however, this was not based on a standardized assessment. Additional information relating to sample characteristics, including sex/gender and race/ethnicity, is provided in Table S5.

#### Intervention delivery

As shown in Table 1, there was considerable variation across studies in their delivery of interventions. In most studies, guidance was provided by the research team (*n* = 18). A teacher provided guidance in thirteen studies and five studies involved guidance from parents. For three remaining studies, one was delivered by school‐based therapists (O’Connor, 2011) and it was unclear who delivered the intervention/guidance in the other two (Li, 2016; Smith, 1978). Intervention exposure varied considerably across studies (see Table 1): 11 had ≤5 (of which eight were limited to a single intervention exposure) and 13 had 6–20. Fourteen studies included ≥21 sessions, most of which were identified as “curriculum‐based approaches” with guided play techniques embedded in everyday classroom experiences. For one study, the number of exposures was unclear (Dejonckheere, 2016).

The interventions included various types of play (see Table S4): most involved pretend play (*n* = 17) and some included more than one type of play, for example, children in Sawyer (2019) engaged in block play and pretend play. For five papers, the type of play was unclear; the focus was on the methods that were used to train the adult, usually a teacher, to guide the children's play.

#### Outcomes measured

A range of learning and developmental outcome measures were identified across studies: language/literacy (early skills, expressive vocabulary, receptive vocabulary, reading skills), numeracy (early skills, spatial/maths language, shape sorting, spatial visualization), executive function/self‐regulation (behavior regulation, task switching, inhibitory control, delay of gratification), socioemotional (prosocial behavior, social competence), visual perception, physical development, science‐learning, creative thinking, and play (exploratory and pretend). The primary outcome and measure used in each study are presented in Table S3.

#### Use of guided play

Sixteen studies explicitly used the term “guided play,” where the conceptualization aligns with the one used in the present review (Weisberg et al., 2013). The remaining 23 studies did not explicitly use the term “guided play,” despite the intervention method fitting the present review's criteria of guided play, though other terms such as, but not limited to, “facilitated play,” “enhanced play,” or “learning through play” were present. As shown in Table S4, a range of guidance methods were used, and common strategies included open‐ended questions, modeling, and hints/prompts. Other methods such as co‐play, setting challenges, and scaffolding were also identified. Further assessment of the delivery of guided play interventions is provided in the narrative synthesis section of this paper.

### Risk of bias in included studies

The Cochrane Risk of Bias Tool (Higgins & Green, 2011) was used to assess the risk of bias across studies (see Figure S1, and for a detailed description see Appendix S4). Almost all studies (*n* = 38) were deemed as having a “high” level of risk, with only one rated as having an “unclear” level (Thibodeau, 2016). Figure S1 illustrates how the domains of random sequence generation, blinding of personnel and outcome assessments, and other pre‐specified bias, are most responsible for the high levels of risk of bias across the studies. Additionally, many studies did not report sufficient information to make confident judgments about allocation concealment, blinding, and/or selective reporting.

### Risk of publication bias across studies

A funnel plot indicated there is some evidence of asymmetry, indicating there is a risk of publication bias (see Figure S2). This was confirmed by a significant Egger's test result (*z* = 2.17, *p* = .03).

### Quantitative synthesis

#### Meta‐analyses of child outcomes

Twelve meta‐analyses were conducted for various child outcomes corresponding to four learning domains: literacy, numeracy, executive function, and socioemotional skills. Most compared guided play and direct instruction, with only one possible comparison for guided play versus free play on numeracy outcomes. Results are presented in Table 2 with a summary below. Forest plots corresponding to each meta‐analyses are provided in Supporting Information (see Figures S3–S14).

**TABLE 2:** Meta‐analytic results

Outcome category	Outcome	Comparison group	*n*	*k*	Effect size	*SE*	*p*‐Value	95% CI	*τ* ^2^	*I* ^2^, %
Literacy	Early literacy skills	Direct instruction	233	2	0.28	0.26	.27	(−0.22, 0.79)	0.08	57.06
	Expressive vocabulary	Direct instruction	628	4	0.21	0.13	.09	(−0.04, 0.46)	0.03	54.87
	Receptive vocabulary	Direct instruction	1564	5	−0.06	0.05	.25	(−0.16, 0.04)	0	0.00
Numeracy	Early maths skills	Direct instruction	1165	2	0.24	0.06	<.001**	(0.12, 0.35)	0	0.00
	Spatial/maths vocabulary	Direct instruction	1214	3	−0.17	0.47	.72	(−1.09, 0.75)	0.61	93.30
	Shape knowledge	Direct instruction	111	3	0.63	0.24	.007**	(0.17, 1.09)	0.04	24.21
	Spatial/maths vocabulary	Free play	137	3	0.93	0.42	.03*	(0.10, 1.75)	0.43	80.70
Executive function	Behavior regulation	Direct instruction	1413	4	−0.03	0.05	.58	(−0.13, 0.08)	0	0.00
	Inhibitory control	Direct instruction	145	3	−0.06	0.17	.71	(−0.39, 0.27)	0	0.00
	Task switching	Direct instruction	132	2	0.40	0.18	.02*	(0.05, 0.74)	0	0.00
Socioemotional	Prosocial behavior	Direct instruction	38	2	1.25	1.41	.38	(−1.51, 4.01)	3.61	90.70
	Social competence	Direct instruction	214	2	0.06	0.14	.68	(−0.21, 0.33)	0	0.00

Note

*k* signifies the number of effect sizes drawn from the equivalent number of studies. *n* signifies the total number of participants included.

* *p* < .05.

** *p* < .01.

There were additional outcome data that could not be entered into meta‐analyses due to substantial differences in the measures used between studies, or because outcomes were limited to single studies. These outcomes correspond to the four learning domains stated above, plus others relating to science, visual perception, physical development, play, and creative thinking. Findings for these additional outcomes are summarized in Appendix S5 and S6. Where possible, the standardized mean difference was calculated and reported, and/or means, standard deviations, and *p*‐values are provided, depending on their availability in study reports. These results should be interpreted with caution as they are more vulnerable to methodological issues (e.g., lack of baseline equivalence between groups).

##### Literacy outcomes

Meta‐analyses identified weak evidence that guided play benefited children's literacy skills more than direct instruction (see Figures S3–S5). Small pooled effects favored guided play for children's early literacy skills, *g* = 0.28, CI: −0.22, 0.79, *p* = .27, *I* ^2^ = 57.06% (combined *n* of two studies = 233) and expressive vocabulary, *g* = 0.21, CI: −0.04, 0.46, *p* = .09, *I* ^2^ = 54.87% (combined *n* of four studies = 628), however the confidence intervals were wide and included no effect. In contrast, there was no evidence of a difference between guided play and direct instruction for receptive vocabulary, *g* = −0.06, CI: −0.16, 0.04, *p* = .25, *I* ^2^ = 0.00% (combined *n* of four studies = 1564).

##### Numeracy outcomes

The results of pooled analyses indicated that guided play, relative to direct instruction, had a small to medium positive effect on two numeracy outcomes (see Figures S6 and S7): early maths skills, *g* = 0.24, CI: 0.12, 0.35, *p* < .001, *I* ^2^ = 0.00% (combined *n* of two studies = 1165) and shape knowledge, *g* = 0.63, CI: 0.17, 1.09, *p* = .007, *I* ^2^ = 24.21% (combined *n* of three studies = 111). However, another pooled analysis of three studies (combined *n* = 1214; see Figure S8) found no difference between guided play and direct instruction on children's spatial and maths vocabulary scores (*g* = −0.17, CI: −1.09, 0.75, *p* = .72, *I* ^2^ = 93.30%). In contrast, a meta‐analyses of three studies (combined *n* = 137; see Figure S9) identified a large effect size favoring guided play versus free play for children's spatial and maths vocabulary (*g* = 0.93, CI: 0.10, 1.75, *p* = .03, *I* ^2^ = 80.70%).

##### Executive function outcomes

Pooled analysis of two studies (combined *n* = 132; see Figure S10) identified a medium effect for guided play compared with direct instruction for task switching (*g* = 0.40, CI: 0.05, 0.74, *p* = .02, *I* ^2^ = 0.00%). Pooled analyses for additional executive function outcomes found no differences between guided play and direct instruction for behavior regulation, *g* = −0.03, CI: −0.13, 0.08, *p* = .58, *I* ^2^ = 0.00% (combined *n* of four studies = 1413; see Figure S11), or for inhibitory control, *g* = −0.06, CI: −0.39, 0.27, *p* = .71, *I* ^2^ = 0.00% (combined *n* of three studies = 145; see Figure S12).

##### Socioemotional outcomes

Results of meta‐analyses found no evidence of differences between guided play and direct instruction for two socioemotional outcomes: prosocial behavior, *g* = 1.25, CI: −1.51, 4.01, *p* = .38, *I* ^2^ = 90.70% (combined *n* of two studies = 38; see Figure S13; note that while the point estimate of the effect size was large, the confidence intervals were very wide, indicating no effect), and social competence, *g* = 0.06, CI: −0.21, 0.33, *p* = .68, *I* ^2^ = 0.00% (combined *n* of two studies = 214; see Figure S14).

### Summary

In summary, meta‐analyses identified significant evidence for guided play having a greater positive effect than direct instruction on early maths skills, shape knowledge, and task switching, and a greater positive effect than free play on spatial vocabulary. Differences were not identified for other numeracy, executive function, literacy, or socioemotional outcomes.

Findings from single studies on these outcomes were mixed and inconclusive, however, when comparing guided play to direct instruction in all outcome domains, single studies identified guided play to have greater positive effects than free play on vocabulary, maths, and some executive function outcomes. Findings on other outcomes (visual perception, physical outcomes, science‐based outcomes, play outcomes) were either limited to a small number of studies or, in the case of play outcomes, were mostly characterized by very small sample sizes, precluding confident inferences about the potential effects of guided play.

#### Moderator analyses

The means and *SD*s from 30 studies were included in moderator analyses. In the following section, *n*s correspond to the number of studies in the analyses. See Table 3 and Figures S15–S25 (in Supporting Information) for a summary of these results. For studies that included *both* direct instruction and free play comparison groups (e.g., Eason, 2020), only the effect for guided play versus direct instruction was entered into the model.

**TABLE 3:** Moderator analysis results

Moderator	Category	*k*	Effect size	*SE*	*p*‐Value	95% CI	*τ* ^2^	*I* ^2^, %
Intervention exposures	1–5	7	0.19	0.30	.52	(−0.39, 0.77)	0.47	78.80
	6–20	11	0.32	0.14	.02*	(0.04, 0.60)	0.11	55.95
	21+	11	0.06	0.10	.53	(−0.14, 0.26)	0.04	45.80
Adult involved	Parent	5	0.24	0.44	.58	(−0.62, 1.10)	0.80	90.53
	Teacher	9	0.19	0.11	.10	(−0.04, 0.41)	0.05	55.11
	Researcher	14	0.24	0.12	.04*	(0.01, 0.47)	0.07	40.94
*N* participants	0–50	15	0.41	0.16	.01**	(0.09, 0.73)	0.22	59.42
	Over 50	15	0.08	0.12	.47	(−0.14, 0.31)	0.13	77.30
Type of comparator	Free play	12	0.68	0.23	.003**	(0.23, 1.12)	0.44	79.61
	Direct instruction	21	0.13	0.11	.26	(−0.10, 0.35)	0.17	77.48
Study design	Randomized controlled trial	19	0.22	0.12	.07	(−0.02, 0.46)	0.18	79.96
	Quasi	11	0.13	0.15	.39	(−0.16, 0.41)	0.10	41.55

Note

*k* signifies the number of effect sizes drawn from the equivalent number of studies.

* *p* < .05.

** *p* < .01.

##### Intensity of intervention exposure

For studies with 1–5 exposures (*n* = 7), there was no evidence of a difference in learning outcomes between guided play and controls (*g* = 0.19, CI: −0.39, 0.77, *p* = .52, *I* ^2^ = 78.80%; see Figure S15). For studies with 6–20 exposures (*n* = 11), a pooled analysis (see Figure S16) found evidence for guided play having a positive effect compared to control conditions (*g* = 0.32, CI: 0.04, 0.60, *p* = .02, *I* ^2^ = 55.95%). No differences were identified for studies including ≥21 exposures (*n* = 11; *g* = 0.06, CI: −0.14, 0.26, *p* = .53, *I* ^2^ = 45.80%; see Figure S17).

##### Adult involved

For studies in which researchers provided guidance (*n* = 14), a pooled analysis identified some evidence of a difference between guided play and controls on learning outcomes (*g* = 0.24, CI: 0.01, 0.47, *p* = .04, *I* ^2^ = 40.94%; see Figure S20). There was no evidence of a difference between guided play and controls for other adults involved, which includes teachers (*n* = 9, *g* = 0.19, CI: −0.04, 0.41, *p* = .10, *I* ^2^ = 55.11%; see Figure S19) and parents (*n* = 5, *g* = 0.24, CI: −0.62, 1.10, *p* = .58, *I* ^2^ = 90.53%; see Figure S18).

##### Number of participants

Pooled analyses found that guided play had a greater positive effect on learning outcomes than controls for studies with <50 participants (*n* = 15; *g* = 0.41, CI: 0.09, 0.73, *p* = .01, *I* ^2^ = 59.42%; see Figure S21), but found no difference for studies with ≥50 participants (*n* = 15; *g* = 0.08, CI: −0.14, 0.31, *p* = .47, *I* ^2^ = 77.30%; see Figure S22).

##### Type of comparator

Pooled analyses indicated a greater positive effect of guided play on learning outcomes versus free play (*n* = 12; *g* = 0.68, CI: 0.23, 1.12, *p* < .01, *I* ^2^ = 79.61%; see Figure S23) but not direct instruction (*n* = 21; *g* = 0.13, CI: −0.10, 0.35, *p* = .26, *I* ^2^ = 77.48%; see Figure S24).

##### Study design

There was weak evidence that guided play benefited children's learning more than the controls for studies that employed an RCT design (*n* = 19; *g* = 0.22, CI: −0.02, 0.46, *p* = .07, *I* ^2^ = 79.96%; see Figure S25), but not a quasi‐experimental design (*n* = 11; *g* = 0.13, CI: −0.16, 0.41, *p* = .39, *I* ^2^ = 41.55%; see Figure S26).

### Qualitative synthesis

#### Narrative synthesis approach

Narrative synthesis was used to address the secondary research question regarding the conceptualization and implementation of guided play with respect to adult guidance and child choice. Using content analysis, similarities and differences were identified across studies in the ways they conceptualized and implemented two key features of guided play–child choice and adult guidance.

**FIGURE 2:**
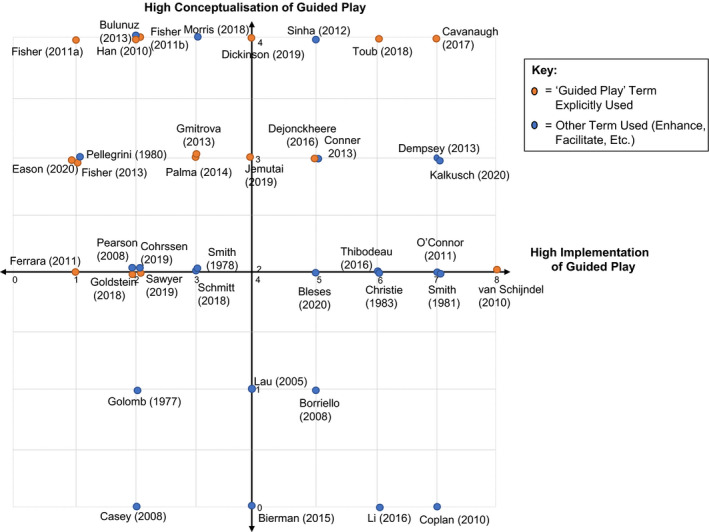
Quadrant illustrating the conceptualization and implementation of guided play studies

Results are presented in a quadrant (see Figure 2): with conceptualization and implementation of guided play plotted along the *Y* axis and *X* axis, respectively. A higher rating of conceptualization was assigned if a study theoretically acknowledged key components of guided play (e.g., child‐led activity, flexible, and sensitive adult guidance). A lower rating was assigned if there was limited acknowledgment of the theoretical value of both adult guidance and child autonomy. Higher ratings of implementation were ascribed to studies that provided children with some degree of choice and flexibility, whilst also involving adult guidance that was sensitive to the children's interests and needs. Lower ratings were assigned if child autonomy was limited and if guidance provided was constrained. Studies with low and high conceptualizations of guided play are shown in the bottom and top quadrants, respectively, and studies low and high levels of implementation of guided play are shown in the left and right quadrants, respectively.

Based on the dispersion of studies in Figure 2, several patterns were highlighted. Conceptualization of guided play differed between studies: some placed value on playful learning without consideration of adult or child roles, while others placed less value on play but acknowledged adult support and self‐directed learning as important factors in children's learning. There were also between‐study variations in the implementation of guided play during the intervention activities, with differences in the amount of autonomy afforded to children and in the amount of adaptive guidance provided by adults. While all included studies met the definition of guided play, the implementation of some interventions was more aligned with the definitions of “direct instruction” or “free play,” particularly when considering child autonomy. For example, there are 13 studies that fall directly on the *X* axis in Figure 2 (i.e., all have been rated as having a medium level of conceptualization). But within this grouping of studies, there is substantial spread along the *X* axis from low to high implementation of guided play. This is reflective of the play spectrum which spans from free play to direct instruction (Zosh et al., 2018). While both van Schijndel (2010) and Ferrara et al. (2011) identified their interventions as guided play and conceptually acknowledged many key features of guided play, implementation differed considerably. Ferrara et al. (2011) is more aligned with “direct instruction” as children were given visual instructions on what to construct during a building task, which restricted their freedom within the activity. In contrast, van Schijndel's (2010) intervention, which aimed to improve children's exploratory play, is more akin to “free play” because children had a choice in whether or not to engage in sandpit play at all, and also in how they interacted with the materials in the sandpit.

Discrepancies also exist within individual studies: while some had a strong conceptualization of guided play, this was not evident in the description of the intervention. For example, children's choice may have been constrained, adults’ behavior may have been scripted (limiting their ability to respond to individual needs), and/or guidance strategies may have been minimal. Conversely, while the theoretical value of guided play was not acknowledged in some studies (low conceptualization), the interventions that were delivered encompassed key features of guided play (high implementation). This was common in studies aiming to improve children's social skills. Play may have been utilized in some interventions because it is a developmentally appropriate medium for social interactions, yet the delivery of the intervention afforded children free choice over their play with adult's responding to children's actions and interests through guidance. This highlights the diversity of researchers which recognize the inherent value of guidance during play for enhancing children's interest and motivation to learn. While some have not explicitly been interested in guided play, in aiming to improve learning outcomes, guided play has been used as a medium for maximizing impact for children, highlighting its instrumental value.

A final observation is that some learning outcomes appear to lend themselves to greater application of guided play features than others. Studies which sought to improve literacy/language and executive function outcomes typically implemented guided play to a higher degree than those assessing numeracy outcomes. This may indicate that maths‐based tasks and measures do not allow for as much child choice and flexible adult guidance as language‐based tasks. Alternatively, maths‐based learning may be better facilitated by teaching methods more in line with direct instruction.

## DISCUSSION

### Summary of evidence

#### Meta‐analyses of child outcomes

The current systematic review aimed to assess the effectiveness of guided play interventions for children's learning and development compared to free play or direct instruction. Thirty‐nine studies were identified for inclusion. Seventeen studies were included in meta‐analyses which produced 12 pooled effect sizes; one for each outcome that was broadly captured by one of four domains: literacy, numeracy, executive function, or socioemotional.

The results of this review provide some evidence that guided play, compared to direct instruction, had a greater positive effect on executive function (task switching only, *g* = 0.40) and maths (including early maths skills, *g* = 0.24, and shape knowledge, *g* = 0.63). However, there were no differences between guided play and direct instruction for literacy or socioemotional outcomes. These results are consistent with previous reviews that found guidance during inquiry‐ or discovery‐based learning benefited science and maths outcomes when compared to traditional teaching (Alfieri et al., 2011; Lazonder & Harmsen, 2016), as did a review of the Tools of the Mind learning through play curriculum (Baron et al., 2017). The overall pattern of these results suggests that guided play may be especially beneficial for maths‐based learning. It could be that the characteristics of guided play are more suited to supporting the development of systematic skills used in maths‐based tasks. For example, guidance techniques, like open‐ended questions or prompts, may guide children toward the next logical step during a maths‐based task. A more nuanced approach of adult guidance may be more effective in supporting learning and development in other areas (e.g., vocabulary, social skills).

The lack of evidence for the benefits of guided play on literacy and socioemotional measures is in contrast with the results of Alfieri et al.’s (2011) review, which identified beneficial effects of guidance on social and verbal outcomes. This may be in part due to limitations in the number and scope of studies of guided play for these outcomes, or may represent a real limitation of guided play approaches for improving these particular outcomes. It is possible that guided play may have more direct effects on outcomes that underpin children's learning, such as children's attitudes and approaches to learning (e.g., motivation, task persistence, and enjoyment). These outcomes were rarely assessed in the studies in this review, despite being salient to children's own descriptions of their early learning experiences (O’Farrelly et al., 2020).

Few studies quantitatively compared guided play and free play, although a single meta‐analysis identified a large effect size favoring the benefits of guided play for spatial vocabulary over free play (*g* = 0.93). However, this result should be interpreted cautiously given the high level of heterogeneity across the studies. Single studies also provided evidence that guided play is better than free play for improving expressive and receptive language, and maths shape knowledge. In addition, single studies that included both direct instruction and free play comparison groups consistently found larger benefits when guided play was compared to free play than when compared to direct instruction (Eason, 2020; Fisher, 2011b; Sinha, 2012; Toub et al., 2018).

#### Moderator analyses

Only two characteristics appeared to have a clear moderating effect on guided play interventions: number of study participants and type of comparator (free play vs. direct instruction). Guided play studies with <50 participants had a significantly greater effect on learning outcomes while no difference was seen for studies with 50 or more participants. However, larger studies (over 50 participants) were also typically “whole curriculum” approaches in which effects of guided play be harder to detect as it is usually used as one of a number of strategies.

Greater effects on learning outcomes were generally seen when guided play was compared to free play rather than direct instruction. It is important to note that studies using free play as a comparator tended to have fewer than 50 participants, making it difficult to disentangle the individual effects of these elements. Given that effects were mostly seen in smaller studies, this is a cause for some caution as larger studies are often more methodologically robust, offering more precise estimates, and greater confidence in the veracity of findings.

There was no clear evidence that the number of sessions in an intervention or the adult involved (teacher, parent, or researcher) moderated the effectiveness of guided play interventions. It may be that guided play is just as valuable to learning both in and out of the classroom environment if it can be effectively implemented (e.g., by parents or a range of school‐based professionals such as teachers, support staff, external therapists).

Other reviews have shown that child age moderates the effectiveness of guidance on learning, with young learners being more influenced by the type of guidance used (Lazonder & Harmsen, 2016). However, this was not examined in the current review due to most studies focusing only on children aged 3–6 years.

#### Narrative synthesis

The conceptualization and implementation of guided play in the included reports were examined using narrative synthesis and considerable variability was found both between and within studies. While many included the key characteristics of guided play (child choice and flexible guidance) in their conceptualization, they were somewhat constrained in their implementation, particularly regarding the amount of choice afforded to children in the play experience. Key features of guided play tended to be more constrained in studies which targeted maths outcomes, for which greater effects were observed in meta‐analyses. A version of guided play that is closer to “playful instruction” on the play spectrum may be more effective for maths outcomes (Zosh et al., 2018). It is possible that studies aiming to improve other outcomes, such as literacy or executive function, may need to constrain play to be more in line with that seen in maths‐focused studies to improve effectiveness.

In the existing literature, there is debate concerning how much guidance and choice is beneficial for learning (Kirschner et al., 2006; Mayer, 2004). Findings from this review suggest that the level of child choice being provided to children is often less than the amount that is conceptually framed as being needed in order to cultivate children's agency, motivation, and curiosity in learning encounters. While definitive conclusions cannot be drawn, the differing degrees of guidance and child choice among studies suggest that the level of child choice may be dependent on the outcome of focus. To further understand the effectiveness of guided play, there is a need for studies that systematically examine the differential effects of guided play with varying levels of flexible adult guidance and free child choice (e.g., a study that compares children in a guided play condition with limited choice to children in a guided play condition with high levels of choice).

Interestingly, numerous studies which implemented interventions that included the characteristics of guided play did not explicitly refer to guided play. The benefits of guided play features may therefore be intuitive and be used instrumentally across several areas to maximize learning opportunities, despite studies not explicitly considering the conceptual basis for why this might be developmentally valuable. Many of the studies which looked to improve socioemotional outcomes, for example, intuitively used features of guided play. This may indicate a field of child development where guided play can be tested more explicitly in future research.

### Strengths and limitations

This is the first systematic review and meta‐analytic study to examine the effects of guided play on a range of learning and developmental outcomes. Narrative synthesis was also used to dissect the delivery of interventions and consider potential implications for future research and education practice.

There are several limitations. First, many of the included studies were assessed as having a high risk of bias due to lack of blinding, lack of using random sequence generation, and/or failure to report sufficient information on allocation concealment and selective reporting (assessed using the Cochrane Risk of Bias Tool). This is in line with findings of other education‐ and play‐based reviews that find even RCT studies to be of lower quality, with consequences for confidence when making inferences from these studies (e.g., Baron et al., 2017; Lillard et al., 2013). It is important to note that this will have been affected by criteria such as the blinding of participants and personnel, which can be difficult to achieve in educational interventions. It was more common for studies to ensure blinding of outcome assessments, which was clear in roughly one‐third of studies. Second, the sample sizes of studies were small with half of papers reporting on samples with <50 participants. Thus, interpretations of results should be made cautiously.

A third limitation is that heterogeneity was particularly high in the moderator analyses. The lack of consistency between studies limits the extent to which results can be confidently generalized (Higgins et al., 2003). However, the main findings from the meta‐analyses (e.g., the effect of guided play on maths‐based outcomes) were low in heterogeneity, thus providing greater confidence in these findings.

There were limitations in the level of detail that could be obtained from the intervention studies. Data on ethnicity and gender/sex were only given in aggregate, limiting our attempts to investigate any impact of these characteristics and the potential generalizability of the findings. Some studies lacked information regarding intervention delivery (i.e., study characteristics), which may have impacted coding and subsequent interpretations. The included reports also lacked detail about the amount of adult‐contact time the control groups received, and whether it was comparable to that of the intervention groups. As social interaction is thought to be associated with learning (Zosh et al., 2017), effect sizes favoring guided play may in part be a result of greater exposure to adult interaction rather than guidance. There was also limited information on the role of play type. This could be an instructive avenue for future research, as different types of play may have specific advantages for learning depending on its context (Smith & Pellegrini, 2013).

Furthermore, sufficient data for meta‐analysis could not be retrieved from some studies and so more evidence may be available for the effectiveness of guided play that could not be included, despite our efforts to seek out additional information. For example, a large scale, curriculum‐based study (Barnett et al., 2008) could not be included in the review due to the challenge of determining how much of the intervention fit with the definition of guided play. A limitation of the review itself is that only post‐intervention data were included. It would be valuable for future studies to look at durability of effects, though few studies included follow‐up data beyond immediate post‐test.

### Implications for theory and research

Further research efforts need to be adequately powered and of a more robust quality in order to derive inferences that are sufficient to guide educational practice. Experimental designs are important to elucidate the benefits of play (Lillard et al., 2013); however, appropriate randomization techniques, reporting of baseline equivalence, and repeated measure designs are needed to provide more confident inferences about the potential benefits of guided play. The results of this review underscore the need for studies that can identify whether the benefits of guided play are domain specific or whether gradients of guided play are better suited to specific outcomes (e.g., whether more constrained experiences are better suited to numeracy outcomes as suggested by the narrative synthesis). To this end the field would benefit from situating guided play experiences within the play spectrum (Zosh et al., 2018). These more refined and deductive hypotheses are well suited to pre‐registered reports and registered trial protocols which could significantly reduce bias, as would the use of pre‐specified and blinded assessments of common outcome measures. Initiatives such as the Child Outcomes Research Consortium and the National Institutes of Health Toolbox are helpful in this regard. Adapting risk of bias tools for greater suitability to educational interventions would also allow for more instructive assessments of study quality.

Studies explicitly testing the mechanisms through which guided play may improve learning, would also provide insight into the outcomes that may benefit most from guided play approaches (Hassinger‐Das et al., 2017). This would allow education interventions to situate guided play components within theories of change, as this can be unclear in studies testing whole curriculum approaches. Guided play may also enhance learning indirectly by impacting children's attitudes to learning and 21st‐century skills (e.g., motivation, persistence, enjoyment, creativity, confidence). When children are supported to undertake and navigate learning experiences that nurture their agency, they may invest more effort and internalize this motivation in their identity as a learner (Deci & Ryan, 2000). Sensitive guidance, may also reduce demands on executive function and working memory, allowing children to engage in more creative processes (see Alfieri et al., 2011) and persist in the face of challenges. If guided play is effective in promoting young children's love of learning, then it may offer cascading effects on learning that go beyond domain‐specific effects on content knowledge that have been the focus of most studies to date.

Widening studies to consider broader age ranges, children with developmental challenges, and low‐income countries/settings would be beneficial for determining the potential values of guided play, especially as greater effects may be seen for at‐risk populations. For example, play‐based learning may be more beneficial for children who struggle to adjust to traditional classroom expectations like sitting and listening for extended time.

### Implications for policy and practice

The review highlights the need for adequately powered, high‐quality research, which provides robust evidence that is firmly placed to inform ECE policy and practice. Nonetheless, there is some evidence that guided play interventions can support maths‐based learning in the classroom. However, questions remain about how key features of guided play (flexible guidance and child choice) are best implemented to foster outcomes. In addition, results comparing guided play to free play suggest that ECE teachers could utilize guidance and support, while children engage in play, to enhance opportunities for academic learning. Initial evidence that the adult delivering the intervention does not impact on outcome, suggests guided play may be suitable for various early learning environments.

Large variations in the delivery of guided play in the review studies highlight the importance of having clear guidance for how adults should implement guided play. This is needed to support the translation of evidence to practice, particularly as ECE educators report wanting greater support in this area (Martlew et al., 2011). Rather than priority being placed on which types of guidance or how much guidance is most effective, education practice may benefit most from supporting teachers to notice and respond to the needs of individual children to guide them effectively. Similarly, there may be value in providing opportunities for children to learn in ways that are of interest to them personally. An understanding of children's own experiences of guided play may also help to identify which features are important for learning experiences.

## CONCLUSION

The review found evidence of an effect of guided play on early maths and related skills but not for other outcomes, including literacy, and socioemotional development. Overall the existing evidence is not of a quantity, quality, or consistency within any one outcome area to allow very confident conclusions to be drawn, as most findings from the meta‐analyses were based on two or three studies in each domain. Nonetheless, the review is instructive in highlighting the diversity across studies in how guided play is conceptualized and operationalized. Greater adoption and further development of theories of change and guided play (including the spectrum of play; Zosh et al., 2017) would provide the field with the unifying framework needed to systematically develop a research program that can better identify where guided play is helpful to children, how it supports learning, and for which outcomes.

## Supporting information

Supplementary Material
