# Supplementary material for: Can guidance during play enhance children’s learning and development in educational contexts? A systematic review and meta‐analysis
Source: Child Dev. 2022 Jan 12;93(4):1162–80. doi: 10.1111/cdev.13730 (PMC9545698; doi:10.1111/cdev.13730)
Supplement: Supplementary file 1 — Supplementary Material [file CDEV-93-1162-s001.docx]

**Supplementary Materials**

**Appendix S1. Definition of Guided Play**

The guided play intervention or activity must meet the following definition of guided play:

- **Child autonomy**: Within a guided play scenario, the child should have an opportunity to have freedom and choice over their own actions and playful behaviour.
- **Adult guided**: either through setting up a play situation or joining a child’s play, an adult should provide guidance using one or more of the following strategies: sensitive hints/prompts, open-ended questions, setting challenges, modelling to guide the child’s attention. Where possible, guidance should be adapted to the individual needs, interests, and understanding of the focus child.
- The play-based task should have a **clear learning goal** which the adult can hold in mind to guide the child towards.

**Characteristics of guided play**

The characteristics of guided play can be broken down into (a) the adult’s role, (b) the child’s role, and (c) the play element. Each are described in turn below.

1. **The adult’s role:** during guided play, an adult (parent/carer/teacher) should act as a guide while holding a clear learning goal in mind. This can be achieved in several ways, and for a play activity to be deemed guided play, an adult must meet at least one of these criteria:

- An adult can **set up a play scenario** with a learning focus in which the child can then play freely. Often this will also involve an element of modelling first, for example, a teacher may first read a book to the children, focusing on target words, and then allow children to play freely with toys related to the story.
- An adult can **provide a challenge** to the child (e.g., asking them to ‘build the tallest tower’ with a focus on improving spatial awareness or language).
- An adult can **provide scaffolding** while a child engages in a play activity, for example, by introducing new ideas, giving meaningful feedback, or using open-ended questions to guide and prompt their thinking (e.g., “What do you think will happen if...?”).

An adult can use **non-verbal guidance** to prompt a child (e.g., guiding direction through pointing and using actions and sounds).

1. Within the guided play scenario, it is important that the **child has freedom and choice** regarding their behaviours. This allows for self-exploration and motivation to increase a child’s learning opportunities. This can occur in several ways:

- The child may be engaging in free play, meaning they have chosen what to play with and how to play with it (an adult can then provide guidance within this).
- The child may be given set toys/resources but then can play with them as they wish. The child may be given a set instruction/goal but has freedom in choosing how they meet this goal.

1. The definition of guided play is concerned more with the role of the adult and child as opposed to the type of play that is occurring. As a result, guided play can take place in several contexts including free play, exploratory play, and role play and can involve a range of play resources such as blocks, sorting toys, themed toys (e.g., farm-related), novel objects, and household items).

The definition provided was informed by Weisberg et al. (2013). Following consultation with an expert panel, the following changes were made to the definition:

- Wording adapted to ensure guided play can be delivered by parents as well as teachers inclusion of non-verbal guidance (with young children in mind specifically).
- Inclusion of distinction between ‘initiated’ (adult sets up activity) and ‘extended’ play (adult joins existing child play).
- Type of play/resources not restricted to account for all playful interactions/experiences.

**Appendix S2. Data Extraction Coding Form**

| **Parent code** | **Child codes** | **Description/guidance** |
| --- | --- | --- |
| **Study design** | | |
| Study design | Randomised Controlled Trial (RCT),  Quasi-experimental,  Not RCT or quasi | RCT – explicitly described as randomised controlled trial or random assignment to conditions  Quasi study – defined as equivalent to RCT (with 2 or more comparison groups) without the randomisation element. This can include repeated measures design as long as outcomes are clearly measured/reported at the midpoint (before groups are switched). |
| **Sample description** | | |
| Child’s age | 1-3 years, 3-6 years, 6-8 years | Based on mean age of child |
| Child development | Typical population,  population with additional needs | This refers to whether the children in the study have any additional learning needs.  If none are mentioned, ‘typical’ should be coded.  Additional needs refers to diagnosed need e.g. Autism. If a study has identified a learning need through a measure e.g. shy or conduct behaviour, still code as ‘typical’ as not a formal diagnosis.  If both apply, select both codes |
| Total number of participants | 0 to 49,  50 to 199,  200+ | Total number of participants when combining the experimental group and control group/s (if there is more than one control, only include participant number from both if both are an appropriate control) |
| **Study context** | | |
| Country | USA, Canada, Australia, Turkey, Portugal, China, Denmark, Netherlands,  South Africa, Kenya | Country in which study was conducted. Tick more than one if applies. |
| Income equality | High inequality (0.5-1.0)  Medium inequality (0.3 – 0.5)  Low inequality (0 – 0.3) | This is determined based on the country in which the study was conducted using the OECD index and GINI index (where OECD missing):  0.5-1.0: South Africa  0.3- 0.5: Turkey, Canada, Australia, USA, China, Kenya, Portugal  0 – 0.3: Netherlands, Denmark |
| Intervention location | Classroom, Home, Childcare centre, Lab, Unclear | Description of where the intervention took place. More than one option can be selected if appropriate.  If unclear, more information should be provided. |
| Adult involved | Teacher,  Parent,  Experimenter/researcher, Other/unclear | This code refers to which adult is described as providing guidance. More than one option can be selected if appropriate.  A range of terms may be used for researcher e.g. student/facilitator (anyone who is research-based)  If other/unclear, more information should be provided. |
| **Description of intervention** | | |
| Number of exposures | 1 to 5,  6 to 20,  21+ exposures (including whole curriculum) | This refers to the total number of days which participants were exposed to the intervention. |
| Key term used | Guided play, Facilitated play, Scaffold play, Supported play  Assist play, Enhance play, Learning through play, Playful learning, Other/unclear | The term does not have to be complete/exact e.g. ‘guidance’ still counts as guided play.  If other/unclear, more information should be provided. |
| Main method of guidance provided | Model  Hint/prompt  Suggestion  Open-ended question  Set challenge  Co-play  Adjust to child’s level/scaffold | Select method of guidance which applies most (select more than one if appropriate).  Papers may not always use these terms explicitly so base on the following definitions:  Model – showing child how to do something (can be physical action or language use) and may be subtle to child  Hint/prompt – wondering aloud ‘I wonder what would happen if..’, or prompting/guiding child’s actions ‘did you want to use the pot or the watering can’. Hints/prompts often subtle and can be non-verbal e.g. pointing.  Suggestion- more directive guidance to pose an option to the child but leave the choice with the child e.g. “shall we start with the puzzle edges?”  Open-ended question – broad questions to help children think about own actions or a learning goal  Set challenge – sets child a challenge with a learning goal e.g. ‘build the tallest tower’, ‘make your own game’  Co-play – adult plays alongside child and immerses self in the play to support social interaction for example or language extension  Adjust to child level/scaffold – noticing child’s interests and cues and providing guidance and increasing or reducing guidance when child does not understand or needs extending.  Unclear/missing (provide info) |
| Child’s level of choice | Choice of activity  Choice of resource within activity  Choice of behaviour within activity  Unclear/missing | This information was extracted in further detail during narrative synthesis coding. This code set provided a starting point regarding how much free choice does the child appear to have in the activity.  Choice of activity –the child has lots of choice e.g., there is no pre-prescribed activity/the child initiates play – gets to choose what they play with and how.  Choice of resource within activity –the child has a good amount of choice within an activity e.g., adult initiates play/sets up activity, but children get to pick which resources they engage with/how engage  Choice of behaviour within activity – the child has limited (but still some) choice e.g., as above except adult also prescribes which resource child uses but child can use this how they wish/behave how they wish  Unclear/missing – if no choice, or none of above categories fit. |
| Description of intervention | Description of intervention | Brief summary of the content of the intervention with anything not covered in above codes e.g., what activity was involved, what did adult do, what did children do, what were the play elements? |
| **Description of control group** | | |
| Control/comparison group - Control group type | Free play  Direct instruction/ business as usual | Free play – comparison group involves children playing or using resources independently without any adult input  Direct instruction – comparison group involves the adult/teacher using instructive methods to direct learning/play. This is primarily traditional teaching methods of the adult talking and the child listening, or the adult telling the child exactly what to do during a task. Business as usual control groups also fall into this category. |
| Control/comparison group - Description of control group content | Description of control group | Brief summary of the content of the control group with anything not covered in above codes e.g., what activity was involved, what did adult do, what did children do, what were the play elements? |
| **Outcome measures** | | |
| Main outcome measure | Academic: Literacy/language, Maths/numeracy, Executive function, Science-related  Socio-emotional: Social skills, Emotions  Physical: Motor skills | More than one can be selected if relevant |
| Quantitative information | Means  Standard deviations | EPPI allows for this information to be input in relation to a chosen outcome and uses *n* to generate an effect size |
| Note. EPPI software used to extract data already records study ID, author/year, type of report, and can generate an effect size, therefore this data did not need to be extracted. | | |

**Appendix S3. Narrative Synthesis Coding Schemes**

**Guided play key features (Weisberg et al. 2013):**

- clear learning goal
- adult guidance (question, model, hint, co-play etc.) while child plays with ideal to adapt to individual children’s level)
- child choice over play (or actions and choices within play)

**Conceptualising guided play**

| High 4 | Paper recognises importance of:   - adult guidance being adapted to child’s individual needs/interests (scaffold) - the value of child having choice, motivation through control of own actions - touch on value of guidance/choice for learning outcomes - importance of play (particularly in relation to learning) - (should acknowledge all to some extent) |
| --- | --- |
| M/H 3 | As above except does not touch on all aspects in lots of detail e.g., may talk a lot about guidance and importance of play, but while acknowledge child’s choice, may not go into as much detail |
| Medium (M) 2 | Recognises value of play (for learning particularly) but perhaps touches less on guidance/choice specifics  OR  Recognises value of adult guidance + child choice but does not acknowledge value of play (essentially touches on some but not all the requirements of guided play) |
| M/L 1 | Falls between medium and low descriptions; may see value in play but no acknowledgment to learning, child choice or adult guidance, or may acknowledge value of guidance and/or child choice to minimal extent. |
| Low (L) 0 | Focus of paper does not relate to value of play, guidance, or child choice. Appears to use play as basis of intervention for reason other than see value of it/doesn’t acknowledge value of play, guidance, or child-led activities e.g., focus may be on social skills but happened to choose play intervention to explore |

**Implementation of guided play- Adult guidance**

| High (H) 4 | Adult uses a variety of guidance methods (question, prompt, hint, extend language, co-play etc.) with clear attempts to adapt to child’s level of need or interest through guidance methods e.g., guidance is clearly in response to child’s actions rather than adult led. |
| --- | --- |
| M/H 3 | As above but to lesser extent: range of guidance used and strong attempts to follow child’s lead or respond specifically to children’s behaviour cues. Following child’s lead occurring to some extent. |
| Medium (M) 2 | Range of guidance methods used but perhaps not very adapted to individual children. Adult may have a brief script to help them but not too constrained (more a guide allowing some flexibility)  OR  One type of guidance used but clear attempts to adapt to individual children or awareness that children may need differing responses. |
| M/L 1 | Elements of adult guidance is scripted and perhaps not much range in guidance type. Not much responsiveness to child actions. Study may increase difficulty of task/guidance over time but not unique to individual children’s needs. |
| Low (L) 0 | Guidance very limited; may only be one type used or generally unclear how guide play. Guidance does not vary at all between children e.g., guidance heavily scripted or guided by a book with no adaptations to individual children. |

**Implementation of guided play - Child choice**

| High (H) 4 | Child has clear opportunity to lead own play actions  e.g., resources provided but child gets to choose how to use and/or clarity in the study that adult involvement is provided based on child’s actions OR no set resources or outcome prescribed, children can choice what and how to engage |
| --- | --- |
| M/H 3 | Children have free choice over play and resources provided, resources pre-selected but range available which gives children choice and use of resources not prescribed by adult. e.g., as above but perhaps actions of adult or reading of story before play may restrict child’s freedom slightly where props/toys reflect the story |
| Medium (M) 2 | Resources and activity prescribed by adult and perhaps initiated by adult but opportunities for children to explore and make choices within this e.g., while co-playing with adult |
| M/L 1 | Children’s play limited to few resources with limited function and actions quite prescribed by adult instruction/guidance or math/board games which have limited variety (only can be played in one way really) |
| Low (L) 0 | Children simply responding to questions/following set instruction while interacting with set resource/task with adult present to extend this further, perhaps only one resource option |

**Appendix S4. Risk of Bias Judgements**

The Cochrane Risk of Bias Tool (Higgins & Green, 2011) was used to assess the risk of bias across studies.

**Random Sequence Generation**

Of the 22 included studies in which randomisation was used, 12 provided sufficient information to assess random sequence generation. Of these, one was considered low risk due to the use of a random draw. 11 studies were considered high risk for several reasons including matching on age, gender, or ability at the point of randomisation or random assignment occurring after initial group assignment occurred. 10 studies were judged to have unclear risk because they did not provide information on randomisation. All 17 quasi-experimental studies were categorised as high risk of bias for random sequence generation given random assignment methods were not used.

**Allocation Concealment**

Allocation concealment was rarely reported in sufficient detail resulting in 21 of the 22 randomised studies being judged to have an unclear risk of bias. One study was reported to be high risk.

**Blinding of Participants and Personnel**

Blinding of personnel delivering guidance during the intervention (whether that be parents, teachers, or members of the research team) lacked clarity with eight studies judged to have unclear risk because they did not report on blinding. A further 30 studies were judged to be high risk because, in delivering the intervention, personnel were identified as un-blinded. Only one study was considered low risk based on its report that all personnel were blinded to the conditions (Thibodeau, 2016).

**Blinding of Outcome Assessment**

A similar issue was seen in judging blinding of outcome assessments, with 11 studies providing insufficient information to make a clear judgement. As these studies did not report on outcome assessment blinding, it is probable that it did not occur, however without having certainty of this information, these papers were judged as having an unclear risk of bias. A further 15 studies were judged to be high risk of bias as assessors were not sufficiently blinded to group allocations. The remaining 13 studies ensured researchers carrying out child assessments or those involved in coding video data were blind to conditions and were therefore judged to be low risk.

**Incomplete Outcome Data**

Primarily due to low dropout rates or reports that attrition was not statistically different across groups, attrition bias was judged to be low risk for 28 studies. A further eight studies were judged as unclear risk due to insufficient information and three were judged to be high risk due to a clear imbalance between groups following attrition.

**Selective Reporting**

While gradually increasing in practice, it is common for education-based studies to not publish a protocol ahead of carrying out research. As a result, only one study could be confidently judged as low risk of bias for selective reporting based on an existing protocol (Morris et al., 2018). Despite not having a protocol, one study was judged to be high risk because the results section did not fairly reflect all the measures outlined in the method section. The remaining 37 studies did not provide sufficient information to make a judgement.

**Other Bias**

To allow for the risk of bias tool to be used with quasi-experimental as well as randomised studies, several other potential biases were pre-specified in the method section of this review for consideration. Based on this, only six papers appeared to be free of other bias and therefore judged to be low risk while an additional two did not provide enough information to make a confident judgement. The remaining 31 studies were identified to be high risk of bias with primary reasons for this judgement including the use of self-developed measures and interventions and only studying children from a single class or school.

**Appendix S5. Quantitative Results: Single Studies**

In this section, results from single studies are reported (i.e., findings related to data from outcomes that were not entered into meta-analyses). Where possible, the standardized mean difference (SMD) scores for guided play versus free play and/or guided play versus direct instruction were calculated and are reported.

**Literacy Outcomes**

***Direct Instruction as Comparator***

Bierman (2015; *n* = 192) and Goldstein (2018; *n* = 39) found no differences between effects of guided play and direct instruction on reading fluency or general vocabulary, respectively. O’Connor (2011) reported that while the direct instruction group showed gains in total language scores, the guided play group did not (*n* = 35; *SMD* = -0.64). Further details are provided below:

- Bierman (2015) measured reading fluency (test of word reading efficiency) which was identified as a literacy outcome but because reading was not measured by any other study, it could not be included in a meta-analysis. An *SMD* of 0.09 was calculated (*M* = 0.05, *SD* = 1.09, *n* = 91; control group: *M* = -0.04, *SD* = 0.92, *n* = 101), and the paper reported no significant effect of intervention on reading fluency.
- Goldstein (2018) measured general vocabulary (WPSSI) which did not fit into the category of expressive or receptive vocabulary and so could not be included in either meta-analysis. A *SMD* of 0.31 was calculated (intervention group: *M* = 9.89, *SD* = 6.26, *n* = 28; control group: *M* = 7.95, *SD* = 5.85, *n* = 11). Despite finding a small effect of the intervention on vocabulary, when controlling for baseline measures, this finding was not reported to be significant.
- O’Connor (2011) reported the following data when measuring total language score (PLS-4); intervention group: *M* = 103, *SD* = 8.02, *n* = 19; control group: *M* = 113, *SD* = 19.9, *n* = 16. We identified *SMD* of -0.64 suggesting direct instruction had a greater effect than guided play on improving total language score. This finding was reported as significant in the paper.
- Smith (1978) reported no group difference (guided play versus direct instruction) in gains of children’s vocabulary and expressive language skills (Reynell Language Scales). Similarly, Smith (1981) found no group differences (guided play versus direct instruction) in gains of children’s language scores (based on the Weschler Preschool and Primary Scale of Intelligence; WPPSI).

***Free Play as Comparator***

Toub (2018, *n* = 167) reported a small but significant effect to both children’s expressive (new word definition test-modified) and receptive vocabulary (PPVT). As was the only study included that included quantitative literacy outcomes when comparing guided play and free play, a meta-analysis was not possible. There were small effect sizes for both expressive (*SMD* = 0.38; intervention group: *M* = 0.59, *SD* = 0.48, *n* = 83; control group: *M* = 0.43, *SD* = 0.41, *n* = 84) and receptive vocabulary (*SMD* = 0.34; intervention group: *M* = 0.6, *SD* = 0.17, *n* = 83; control group: *M* = 0.54, *SD* = 0.18, *n* = 84).

Conner (2013) reported that children in the intervention, but not control, group showed gains in receptive and expressive vocabulary (based on the Preschool Language Scale). Both the groups demonstrated improvements on a vocabulary assessment of comprehension and expression of target words, but children in the guided play condition showed greater gains (effect size and significance level were not reported for this outcome).

**Numeracy Outcomes**

***Direct Instruction as Comparator***

Several numeracy outcomes could not be combined for meta-analyses. In these studies, no differences were reported between guided play and direct instruction on various outcomes, including spatial complexity of drawings (Sawyer, 2019; *n* = 37), maths problem solving, number counting, or number naming (Cohrssen, 2019; *n* = 60). Mixed findings were reported by Casey (2008; *n* = 100): there were small effect sizes favouring guided play for two spatial measures, block building (*SMD* = 0.30) and spatial visualisation (*SMD* = 0.35), however there was a medium effect size favouring direct instruction for mental rotation (*SMD* = -0.59). Further details are provided below:

- Cohrssen (2019) reported on three numeracy outcomes which could not be grouped in a meta-analysis due to lack of overlap with other studies. For applied math problems (Woodcock-Johnson), a small effect size was found favouring direct instruction over guided play (*SMD* = -0.21; intervention group: *M* = 11.3, *SD* = 4.83, *n* = 22; control group: *M* = 12.3, *SD* = 5.08, *n* = 38). Whereas measures of number counting and number naming saw medium to large effect sizes favouring guided play over direct instruction. For number counting, a *SMD* of 0.65 was calculated (intervention group: *M* = 6.97, *SD* = 2.57, *n* = 22; control group: *M* = 5.4, *SD* = 2.26, *n* = 38). For number naming, a *SMD* of 0.48 was calculated (intervention group: *M* = 5.9, *SD* = 3.08, *n* = 22; control group: *M* = 4.53, *SD* = 2.67, *n* = 38). None of these effects were reported to be significant.
- Sawyer (2019) measured spatial complexity of children’s drawings (CAT). We identified an *SMD* score of -0.06, supporting the reported finding of no difference in effect between guided play and direct instruction on spatial complexity (intervention group: *M* = 2.91, *SD* = 1.78, *n* = 28; control group: *M* = 3.03, *SD* = 2.15, *n* = 9).
- Casey (2008) compared guided play and direct instruction in relation to spatial skills with results varying depending on school location and measure of spatial skills used. This variety meant it was not possible to select one outcome for inclusion in the meta-analyses. School locations differed somewhat in terms of the ethnicity of pupils and household income, but these differences were not reported to be significant. For one school, a small effect was seen to favour guided play for block building scores (*g* = 0.30) and spatial visualisation (*g* = 0.35) but no difference in effect was seen for mental rotation score (*g* = -0.19). For the other school, no effect was seen between guided play and direct instruction for block building (*g* = 0.14) or spatial visualisation (*g* = 0.12), but a medium effect was seen to favour direct instruction over guided play for mental rotation score (*g* = -0.59).

***Free Play as Comparator***

Only one study examined children’s shape sorting performance: Fisher (2011b) reported greater benefits for guided play versus free play (on a typical shape sorting task; *SMD* = 1.27; intervention group: *M* = 0.96, *SD* = 0.10, *n* = 12; control group: *M* = 0.71, *SD* = 0.25, *n* = 12).

**Executive Function Outcomes**

***Direct Instruction as Comparator***

One study measured performance on a delay of gratification task (snack delay task). Sinha (2012) reported no group difference between guided play and direct instruction (*SMD* = 0.07; intervention group: *M* = 25.10, *SD* = 2.40, *n* = 10; control group: *M* = 24.90, *SD* = 3.10, *n* = 11).

***Free Play as Comparator***

Only one study assessed the impact of guided play relative to free play on executive function measures, with mixed findings depending on the measure. Sinha (2012), reported greater positive effects for guided versus free play to children’s delayed gratification (*SMD* = 0.23; intervention group: *M* = 25.1, *SD* = 2.4, *n* = 10; control group: *M* = 24.4, *SD* = 3.3, *n* = 10) and inhibitory control (Stroop task; *SMD* = 0.37; intervention group: *M* = 201, *SD* = 83.4, *n* = 10; control group: *M* = 231, *SD* = 70.3, *n* = 10). However, there was no group difference for behaviour regulation (HTKS task; *SMD* = 0.08; intervention group: *M* = 22.00, *SD* = 13.40, *n* = 10; control group: *M* = 20.90, *SD* =13.80, *n* = 10).

**Socioemotional Outcomes**

***Direct Instruction as Comparator***

Several domains of socioemotional development could not be combined in meta-analyses. Guided play had a significantly greater positive effect than direct instruction on children’s time spent in peer interactions (Li, 2016; *n* = 16), however, no significant effects were reported for social disruption, disconnection, or interaction (O’Connor, 2011; *n* = 35), or speech performance (Li, 2016). Other studies reported mixed results depending on which measures of social interaction and emotional competence were used (Coplan, 2010; Goldstein, 2018). Additional details are provided below:

- Goldstein (2018) assessed a range of socioemotional outcomes that could not be grouped for meta-analyses. There was a small effect size favouring guided play (versus direct instruction) for an emotion matching measure (*SMD* = 0.22), but the effect sizes for measures of comforting behaviours (*SMD* = 0.02) and social interactions (*SMD* = -0.10) suggested no group differences. In contrast, there were small effect sizes favouring direct instruction over guided play for measures of altruism (*SMD* = -0.38), helping behaviours (*SMD* = -0.26), and emotional distress (*SMD* = -0.26). The paper did not report significance values for these outcomes.
- Li (2016) reported on two socioemotional outcomes that could not be included in a meta-analyses. The study reported that guided play had a significantly greater effect than direct instruction on time spent in peer interactions, and is supported by a large effect size (*SMD* of 1.29; intervention group: *M* = 0.27, *SD* = 0.15, *n* = 8; control group: *M* = 0.09, *SD* = 0.11, *n* = 8). A large effect size favouring guided play was also calculated for social communicative competence, however, the study reported this result as non-significant (*SMD* = 1.36; intervention group: *M* = 2.42, *SD* = 0.35, *n* = 8; control group: *M* = 1.20, *SD* = 1.15, *n* = 8).
- O’Connor (2011) reported three outcomes which could not be grouped for meta-analyses, including social disruption, social disconnection, and social interaction. Due to baseline imbalance between the groups on these outcomes (social disruption and social disconnection), post-test effect sizes were not calculated. The authors reported no group differences on social disruption and social disconnection when baseline scores were accounted for in the analysis. Group differences on social interaction were not reported.
- Coplan (2010) reported included two measures that could not be grouped in meta-analyses. The study found that guided play versus direct instruction had a significantly larger effect on reticent wariness (*SMD* = -0.69; intervention group: *M* = -0.35, *SD* = 0.63, *n* = 11; control group: *M* = 0.35, *SD* = 1.21, *n* = 11). In contrast, direct instruction impacted a measure of anxious behaviour more than guided play (*SMD* = -0.71; intervention group: *M* = 1.29, *SD* = 0.33, *n* = 11; control group: *M* = 1.59, *SD* = 0.47, *n* = 11).
- Bierman (2015) was the only study to measure aggression (as a control variable) and reported that guided play versus direct instruction did not significantly impact children’s teacher-rated aggression scores (*SMD* = 0.15; intervention group: *M* = 2.14, *SD* = 0.69, *n* = 91; control group: *M* = 2.03, *SD* = 0.73, *n* = 101).

***Free Play as Comparator***

The results of Pearson (2008) provided no convincing evidence that guided play benefits children’s social competence and hope relative to free play (Pearson 2008; *n* = 32), and Lau (2005; *n* = 36) reported a small but non-significant effect favouring free play versus guided play on a measure of social skills. Additional details are provided below:

- Pearson (2008) assessed children’s teacher- and parent-rated social competence. There was no group difference for teacher-reported social competence (*SMD* = 0.09), but a small effect size favoured free play children for parent-reported social competence (*SMD* = -0.32; intervention group: *M* = 41.80, *SD* = 5.74, *n* = 16; control group: *M* = 44.1, *SD* = 8.65, *n* = 16). Pearson (2008) also measured children’s hope via child-, teacher-, and parent-reports. Calculated effect sizes indicated no difference between guided play and free play on child- or parent-rated hope scores (*SMD’*s were -0.08 and 0.02, respectively), though a medium effect size favoured guided play over free play for teacher-reported hope (*SMD* = 0.41; intervention group: *M* = 16.80, *SD* = 1.73, *n* = 16; control group: *M* = 15.90, *SD* = 2.14, *n* = 16). However, the effect was report as non-significant when controlling for baseline scores.
- Lau (2005) measured teacher-reported social skills. Free play had a small, positive but non-significant impact on children’s peer interactions compared to guided play (*SMD* = -0.23; intervention group: *M* = 6.22, *SD* = 8.91, *n* = 18; control group: *M* = 8.17, *SD* = 7.91, *n* = 18).

**Appendix S6. Additional Outcomes and Results**

**Academic Literacy**

**Academic Numeracy**

The results reported in Fisher (2013) include the participants from Fisher (2011b) plus a further 23 participants, however, the results of the additional participants (for whom outcome assessment blinding was used) were not reported separately from the original results and so could not be included in meta-analysis. Nonetheless, the paper reports that guided play has a significantly greater effect than direct instruction and free play on improving typical shape knowledge (*p* < .001). Smith (1981) assessed arithmetic and geometric design using the WPPSI. Results were reported for the two schools (schools A and B) and the intervention and control groups within each school. Children in both groups and both schools improved their performance on both WPPSI measures. The paper reports that the children in the play tutored group in School A scored significantly higher on the measure of geometric design compared to the control group (*p* > 0.05). However, the same results were not found in school B or on the measure of arithmetic.

**Academic Science-based**

Three studies compared the impact of guided play and direct instruction on science-based outcomes. Children taking part in guided play interventions outperformed direct instruction controls on measures of conservation judgement (Golomb, 1977; *n* = 30; *p* < 0.05, nonparametric test) and of various other scientific measures related to concepts such as gravity and magnets (Bulunuz, 2013; *n* = 26; all *p*s < 0.001). In contrast, a relatively large-scale, curriculum-based study conducted by Morris (2018; *n* = 300) reported no group differences in children’s learning of health/wellbeing and sustainability (based on a composite score from the Healthy Eating and Sustainability assessment). Note that means, SDs, and *p* values were not provided in the report for this analysis.

**Visual Perception**

Jemutai (2019; *n* = 77) examined the impact of guided play on children’s visual perception in two settings: South Africa and Kenya. Although the mean score changes were higher in both intervention groups compared to direct instruction (South Africa: guided play [*M* change = 3.78, *SD* = 6.03] versus the direct instruction [*M* change = 1.95, *SD* = 5.56], *p* = 0.17; Kenya: guided play [*M* change = 6.68, *SD* = 6.79] versus direct instruction [*M* change = 3.70, *SD* = 6.91], *p* = 0.09), the reported results did not meet conventional levels of significance.

**Physical Development**

Palma (2014; n = 71) reported benefits to children’s gross motor development (Test of Gross Motor Development (TGMD-2; Ulrich, 2000) for guided play versus both free play (*p* = 0.02) and direct instruction (*p* = 0.008). However, means and SDs associated with this outcome measure were not reported in the paper.

**Play-based**

Several studies evaluated the impact of guided play on children’s pretend play and exploratory play behaviours. However, data related to these outcomes were not entered into meta-analyses due to inconsistencies in the comparison groups and outcome measures used across studies. Furthermore, they did not align with the pre-specified primary outcome categories. Note that most, but not all, of the studies examining play outcomes had markedly small sample sizes.

Studies comparing the effects of guided play versus direct instruction on children’s play-related outcomes, reported mixed findings. Dejonckheere (2016) found that guided play increased children’s exploratory play behaviours more than direct instruction (*SMD* = 0.80; *n* = 57), and while Kalkusch (2020) reported that guided play increased the quality of children’s pretend play more than direct instruction (*SMD* = 0.93; *n* = 54), another study did not (Christie, 1983; *SMD* = 0.04; *n* = 17). O’Connor (2011; *n* = 35) assessed the impact of guided play versus direct instruction to the following measures: number of object substitutions, number of imitations, and percentage of pretend play. However, there were group differences at baseline, meaning that it was not possible to calculate reliable estimates of group differences for all measures. After controlling for baseline differences for one measure – number of object substitutions, the authors did not report a group difference at post-test.

Smith (1978) reported group by time interactions indicating larger increases in fantasy play activity for guided play than direct instruction (however, no further comparisons were made). This study also reported that children in both groups engaged in more social play, however, there was no differential increase for the children who engaged in guided play.

Smith (1981) found overall increases in the complexity of children’s fantasy play (engaging in guided play or direct instruction), but there were no group differences in these gains. Smith (1981) also reported that there was an effect of group on social participation for the children in school A, however this is likely because the social participation scores of children in the control group decreased between pre- and post-test. There was also a group by time interaction at school B, suggesting increased social participation in the play tutored group. However, children in this group initially scored lower on social participation at pre-test. Finally, there was a group by time interaction reported for both schools for large social group play. However, this effect is again likely driven by a decrease in play scores of the direct instruction group between pre- and post-test.

The impact of guided play versus free play on children’s play-based outcomes was also assessed. van Schijndel (2010; *n* = 28) found that guided play had a greater positive effect on children’s engagement in exploratory play behaviours than free play (*SMD* = 0.38), and Dempsey (2013; *n* = 9) found that guided play increased the amount of time children spent engaging in pretend play more than free play (*SMD* = 1.93; *p* = .048, using a non-parametric test), however Conner (2013; *n* = 10) did not (*SMD* = 0.05). Gmitrova (2013) observed more play behaviours in children that engaged in a guided play intervention session compared to free play controls, however this was not sustained at follow-up.

**Creative Thinking**

Pelligrini (1980, *n* = 16) found that children were significantly more likely to generate creative reasons for object use (i.e., associative fluency) during guided play than during free play or direct instruction (both *p*s < .002). In another study, creative thinking was assessed across three sub-dimensions: there were no benefits of guided play versus direct instruction for originality (*SMD* = -0.07), and small (*SMD* = -0.33) and large (*SMD* = -0.86) negative effects on fluency and imagination, respectively (Christie, 1983; *n* = 17). Finally, both Smith (1978) and Smith (1981) found no group differences between guided play and direct instruction in gains on a creative thinking task.

**Other**

Smith (1978) examined gains in children’s preschool readiness using the Caldwell Co-operative Preschool Inventory, but reported no group difference between guided play and direct instruction.

**Appendix S7. References of studies included in the review**

Bierman, K. L., Welsh, J. A., Heinrichs, B. S., Nix, R. L., & Mathis, E. T. (2015). Helping Head Start Parents Promote Their Children’s Kindergarten Adjustment: The Research-Based Developmentally Informed Parent Program. *Child Development, 86,* 1877-1891. DOI: 10.1111/cdev.12448

Bleses, D., Jensen, P., Slot, P., & Justice, L. (2020). Low-cost teacher-implemented intervention improves toddlers’ language and math skills. *Early Childhood Quarterly, 53*, 64-76. DOI: 10.1016/j.ecresq.2020.03.001

Borriello, G. A., & Liben, L. S. (2018). Encouraging Maternal Guidance of Preschoolers’ Spatial Thinking During Block Play. *Child Development, 89,* 1209-1222. DOI: 10.1111/cdev.12779

Bulunuz, M. (2013). Teaching science through play in kindergarten: does integrated play and science instruction build understanding? *European Early Childhood Education Research Journal, 21,* 226-249. DOI: 10.1080/1350293X.2013.789195

Casey, B. M., Andrews, N., Schindler, H., Kersch, J. E., Samper, A., & Copley, J. (2008). The Development of Spatial Skills through Interventions Involving Block Building Activities. *Cognition and Instruction, 26,* 269-309. DOI: 10.1080/07370000802177177

Cavanaugh, D., Clemence, K., Teale, M., Rule, A., & Montgomery, S. (2017). Kindergarten scores, storytelling, executive function and motivation improved through literacy-rich guided play. *Early Childhood Educational Journal, 45*, 831-843. DOI: 10.1007/s10643-016-0832-8

Christie, J. F. (1983). The effects of play tutoring on young children’s cognitive performance. *Journal of Educational Research, 76*, 326–330. DOI: 10.1080/00220671.1983.10885477

Cohrssen, C., & Niklas, F. (2019). Using mathematics games in preschool settings to support the development of children's numeracy skills. *International Journal of Early Years Education, 27,* 322-339. DOI: 10.1080/09669760.2019.1629882

Conner, J., Kelly-Vance, L., Ryalls, B., & Freihe, M. (2014). A play and language intervention for two-year-old children: Implications for improving play skills and language. *Journal of Research in Childhood Education, 28,* 221-237. DOI: 10.1080/02568543.2014.883452

Coplan, R.J., Schneider, B. H., Matheson, A., & Graham, A. (2010). ‘Play skills’ for shy children: development of a Social Skills Facilitated Play early intervention program for extremely inhibited pre-schoolers. *Infant and Child Development, 19*, 223-237. DOI: 10.1002/icd.668

Dejonckheere, P. J. N., De Wit, N., Van de Keere, K., & Vervaet, S. (2016). Exploring the classroom: Teaching science in early childhood. *International Electronic Journal of Elementary Education, 8,* 537–558. DOI: 10.12973/eu-jer.5.3.149

Dempsey, J., Kelly-Vance, L., & Ryalls, B. (2013). The effect of a parent training program on children’s play. *International Journal of Psychology: A Biopsychosocial Approach, 13,* 117-138. DOI: 10.7220/1941-7233.13.6

Dickinson, D. K., Collins, M. F., Nesbitt, K., Toub, T. S., Hassinger-Das, B., Hadley, E. B., Hirsh-Pasek, K., & Golinkoff, R. M. (2019). Effects of Teacher-Delivered Book Reading and Play on Vocabulary Learning and Self-Regulation among Low-Income Preschool Children. *Journal of Cognition and Development, 20*, 136-164. DOI: 10.1080/15248372.2018.1483373

Eason, S. H., & Ramani, G. B. (2020). Parent-Child Math Talk About Fractions During Formal Learning and Guided Play Activities. *Child Development, 91,* 546-562. DOI: 10.1111/cdev.13199

Ferrara, K., Hirsh-Pasek, K., Newcombe, N., Golinkoff, R., & Shallcross Lam, W. (2011) Block Talk: Spatial Language during Block Play. *Mind, Brain, and Education, 5*, 143-151. DOI: 10.1111/J.1751-228X.2011.01122.X

Fisher, K. R. (2011a; 2011b). Exploring the mechanisms of guided play in preschoolers' developing geometric shape concepts. *Dissertation Abstracts International: Section B: The Sciences and Engineering.*

Fisher, K., Hirsh-Pasek, K., Newcombe, N., & Golinkoff, R. (2013). Taking shape: supporting pre-schooler's acquisition of geometric knowledge through guided play. *Child Development, 84,* 1872-1878. DOI: 10.1111/cdev.12091

Gmitrova, V. (2013). Teaching to play performing a main role - effective method of pretend play facilitation in preschool-age children. *Early Child Development and Care*, *183*, 1705–1719. DOI: 10.1080/03004430.2012.746970

Goldstein, T. G., & Lerner, M. D. (2018). Dramatic pretend play games uniquely improve emotional control in young children. *Developmental Science, 21*. DOI: 10.1111/desc.12603

Golomb, C., & Cornelius, C. B. (1977). Symbolic play and its cognitive significance.  *Developmental Psychology, 13*, 246-252. DOI: 10.1037/0012-1649.13.3.246

Han, M., Moore, N., Vukelich, C., & Buell, M. (2010). Does play make a difference? Effects of play intervention on at-risk preschoolers’ vocabulary learning. *American Journal of Play, 3*, 82–105.

Jemutai, S., & Webb, P. (2019). Effects of a 6 Brick Duplo Block guided pay intervention on pre-literate learners' visual perception. *South African Journal of Childhood Education, 9*. DOI: 10.4102/sajce.v9i1.634

Kalkusch, I., Jaggy, A. K., Burkhardt Bossi, C., Weiss, B., Sticca, F., & Perren, S. (2020). Promoting Social Pretend Play in Preschool Age: Is Providing Roleplay Material Enough? *Early Education and Development,* 1–17. DOI: 10.1080/10409289.2020.1830248

Lau, C., Higgins, K., Gelfer, J., Hong, E., & Miller, S. (2005). The Effects of Teacher Facilitation on the Social Interactions of Young Children during Computer Activities. *Topics in Early Childhood Special Education 25*, 208-217. DOI: 10.1177/02711214050250040201

Li, Y., Coplan, R. J., Wang, Y., Yin, J., Zhu, J., Gao, Z., & Li, L. (2016). Preliminary Evaluation of a Social Skills Training and Facilitated Play Early Intervention Programme for Extremely Shy Young Children in China. *Infant and Child Development, 25*, 565-574. DOI: 10.1002/icd.1959

Morris, H., Edwards, S., Cutter-Mackenzie, A., Rutherford, L., Williams-Smith, J., & Skouteris, H. (2018). Evaluating the Impact of Teacher-designed, Wellbeing and Sustainability Play-based Learning Experiences on Young Children's Knowledge Connections: A Randomised Trial. *Australasian Journal of Early Childhood, 43*, 33- 42. DOI: 10.23965/AJEC.43.4.04

O'Connor, C., & Stagnitti, K. (2011). Play, Behaviour, Language and Social Skills: The Comparison of a Play and a Non-Play Intervention within a Specialist School Setting. *Research in Developmental Disabilities: A Multidisciplinary Journal, 32*, 1205- 1211. DOI: 10.1016/j.ridd.2010.12.037

Palma, M. S., Pereira, B., & Valentini, N. C. (2014). Guided Play and Free Play in an Enriched Environment: Impact on Motor Development. *Motriz-revista De Educacao Fisica*, 20, 177-185. DOI: 10.1590/S1980-65742014000200007

Pearson, B. L. (2008). Effects of a Cognitive Behavioral Play Intervention on children's hope and school adjustment. *ProQuest Information & Learning*.

Pellegrini, A. D., & Greene, H. (1980). The Use of a Sequenced Questioning Paradigm to Facilitate Associative Fluency in Preschoolers. *Journal of Applied Developmental Psychology, 1,* 189-200. DOI: 10.1016/0193-3973(80)90008-8

Sawyer, J. E., & Goldstein, T. (2019). Can Guided Play and Storybook Reading Promote Children’s Drawing Development? *Empirical Studies of the Arts, 37*, 32-59. DOI: 10.1177/0276237418777946

Schmitt, S. A., Korucu, I., Napoli, A. R., Bryant, L. M., & Purpura, D. J. (2018). Using block play to enhance preschool children’s mathematics and executive functioning: A randomized controlled trial. *Early Childhood Research Quarterly, 44*, 181-191. DOI: 10.1016/j.ecresq.2018.04.006

Sinha, J. M. (2012). The Land of Make-Believe: Using Sociodramatic Play to Increase Kindergartners' Self-Regulatory Abilities. *ProQuest LLC*.

Smith, P. K., Dalgleish, M., & Herzmark, G. (1981). A Comparison of the Effects of Fantasy Play Tutoring and Skills Tutoring in Nursery Classes. *International Journal of Behavioral Development, 4*, 421–441. DOI: 10.1177/016502548100400403

Smith, P. K., & Syddall, S. (1978). Play and Non-Play Tutoring in Pre-School Children: Is It Play or Tutoring Which Matters? *British Journal of Educational Psychology, 48*, 315–325. DOI: 10.1111/j.2044-8279.1978.tb03017.x

Thibodeau, R. M., Gilpin, A. T., Brown, M. M., & Meyer, B. A. (2016). The effects of fantastical pretend-play on the development of executive-functions: An intervention study. *Experimental Child Psychology, 145*, 120-138. DOI: 10.1016/j.jecp.2016.01.001

Toub, T., Hassinger-Das, B., Nesbitt, K., Ilgave, H., Weisberg, D., Hirsh-Pasek, K., Golinkoff, R., Nicolopoulou, A., & Dickinson, D. (2018). The language of play: developing preschool vocabulary through play following shared book-reading. *Early Childhood Research Quarterly, 4*, 1-17. DOI: 10.1016/j.ecresq.2018.01.010

van Schijndel, T. J. P., Singer, E., van der Maas, H. L. J., & Raijmakers, M. E. J. (2010). A sciencing programme and young children's exploratory play in the sandpit. *European Journal of Developmental Psychology,* 7, 603-617. DOI: 10.1080/17405620903412344

| **Table S1.** *Search Term Strategy – The Populations, Interventions, and Outcomes.* | | | |
| --- | --- | --- | --- |
| **Question components and a selection of relevant terms** | **Type of terms** | | **Boolean operator** |
|  | **Free** | **MeSH** |  |
| **The populations: young children under the age of 8** | | | |
| No search is performed to capture population |  |  |  |
| **The interventions: guided play - note: when searching in Scopus and web of science, all intervention terms need to be contained within speech marks e.g. “guide* play” “adult guide*”** | | | |
| 1. guide* play | x |  |  |
| 1. facilitate* play | x |  |  |
| 1. enhance* play | x |  |  |
| 1. scaffold* play | x |  |  |
| 1. assist* play | x |  |  |
| 1. support* play | x |  | OR (captures *intervention*) |
| 1. play based learning | x |  |  |
| 1. play-based learning | x |  |  |
| 1. learning through play | x |  |  |
| 1. purposeful play | x |  |  |
| 1. or/ 1-10 |  |  |  |
| 1. adult guide* | x |  |  |
| 1. parent guide* | x |  |  |
| 1. teacher guide* | x |  |  |
| 1. adult scaffold* | x |  |  |
| 1. parent scaffold* | x |  |  |
| 1. teacher scaffold* | x |  |  |
| 1. adult facilitate* | x |  |  |
| 1. parent facilitate* | x |  |  |
| 1. teacher facilitate* | x |  | OR (captures *intervention*) |
| 1. adult assist* | x |  |  |
| 1. parent assist* | x |  |  |
| 1. teacher assist* | x |  |  |
| 1. adult support* | x |  |  |
| 1. parent support* | x |  |  |
| 1. teacher support* | x |  |  |
| 1. adult enhance* | x |  |  |
| 1. parent enhance* | x |  |  |
| 1. teacher enhance* | x |  |  |
| 1. or/ 12-29 |  |  |  |
| 1. play | x |  |  |
| 1. 30 AND 31 |  |  | AND (combines selected intervention terms with ‘play’) |
| 1. 11 OR 32 |  |  | OR (captures *intervention*) |
| **The outcomes** | | | |
| No search is performed to capture outcomes |  |  |  |

| **Table S1 Continued.** *Search Term Strategy - The Study Designs (Broken Down for Each Database).* | | | | | | | | | | | |
| --- | --- | --- | --- | --- | --- | --- | --- | --- | --- | --- | --- |
| **Database: ERIC** | **Type of terms** | | **Database: BEI** | **Type of terms** | | **Databases:**  **PsycINFO and PsycARTICLES** | **Type of terms** | | **Databases:**  **Child Development & Adolescent studies and Scopus and Web of Science** | **Type of terms** | |
|  | Free | MeSH |  | Free | MeSH |  | Free | MeSH |  | Free | MeSH |
| 1. DE “Comparative Analysis” |  | x | 1. DE “EXPERIMENTAL design” |  | x | 1. DE “Experiment Controls” |  | x | 1. “randomised controlled trial” | x |  |
| 1. DE "Experimental Groups" |  | x | 1. DE “CONTROL groups” |  | x | 1. DE “Quasi Experimental Methods” |  | x | 1. “randomized controlled trial” | x |  |
| 1. DE "Control Groups" |  | x | 1. “randomised controlled trial” | x |  | 1. DE "Randomized Controlled Trials" |  | x | 1. “rct” | x |  |
| 1. DE "Quasiexperimental Design" |  | x | 1. “randomized controlled trial” | x |  | 1. DE “Experimental Design” |  | x | 1. “random” | x |  |
| 1. DE "Randomized Controlled Trials" |  | x | 1. “rct” | x |  | 1. DE “Intervention” |  | x | 1. “non-random” | x |  |
| 1. DE “Intervention” |  | x | 1. “non-random” | x |  | 1. DE “Random Sampling” |  | x | 1. “nonrandom” | x |  |
| 1. “experimental condition” | x |  | 1. “control condition” | x |  | 1. “control condition” | x |  | 1. “experimental condition” | x |  |
| 1. “random” | x |  | 1. “nonrandom” | x |  | 1. “random” | x |  | 1. “intervention” | x |  |
| 1. “control condition” | x |  | 1. “experimental condition” | x |  | 1. “experimental condition” | x |  | 1. “control condition” | x |  |
| 1. “non-random” | x |  | 1. “intervention” | x |  | 1. “non-random” | x |  | 1. “quasi” | x |  |
| 1. “nonrandom” | x |  | 1. “quasi” | x |  | 1. “nonrandom” | x |  | 1. or/ 34 - 43 |  |  |
| 1. or/ 34 - 44 |  |  | 1. “random” | x |  | 1. or/ 34 - 44 |  |  | 1. 33 AND 44 |  |  |
| 1. 33 AND 45 |  |  | 1. or/34 - 45 |  |  | 1. 33 AND 45 |  |  |  |  |  |
|  |  |  | 1. 33 AND 46 |  |  |  |  |  |  |  |  |

| **Table S2.** *Studies Excluded due to Lack of Information/Access* | | | |
| --- | --- | --- | --- |
| Study ID | Main outcome(s) | Brief study/ intervention description | Reason for exclusion |
| Barnett (2008) | Literacy, language, and social behaviour | RCT of Tools of the mind curriculum intervention | Not enough information available to determine how much of intervention related to guided play and whether control group fit into category of ‘direct instruction’ or not given it was another curriculum-based intervention.  Contact made with authors – no response |
| Feitelson (1973) | Creative thinking and play | Play tutoring of children’s thematic play was compared with free-play. | Not enough information available regarding the play tutor’s role in the intervention activity (e.g., whether the play tutor guided children or directed them). Contact was made with an author, but additional details could not be acquired to determine eligibility for inclusion. |
| Fleming (2005) | Joint engagement | Training intervention for parents to improve parent-child interactions using scaffolding during play | This paper could not be accessed by the review authors. It is likely the paper would have been excluded as it appears not to have a control group, but further access would be needed to make this judgement. |
| Gallegos (1983) | Academic readiness skills | A comparison between a learning through play curriculum and direct instruction classrooms | This paper could not be accessed by the review authors. It is likely the paper would have been excluded as it appears to compare schools with existing play-based curriculums rather than implementing a new intervention, but further access would be needed to make this judgement. |
| Greene (2018) | Preschool readiness, social skills | Quasi-experimental study using a ‘Living Arts’ intervention, compared to business-as-usual (head start classrooms) | Content of the intervention was unclear and so interpreting the amount of guided play was not possible. Contact made with authors – no response. |
| Popoola (2014) | Early numeracy skills | Quasi-experimental study using the play-way method | Content of the intervention was unclear and so interpreting amount of guided play was not possible. Contact made with authors – no response. |
| Yount (2011) | Play and language skills | Play intervention using stories and toy sets to reinforce language and play | This dissertation could not be accessed by the review authors. It is likely the paper would have been included in the review, but further access would be needed to make this judgement. |

| **Table S3.** *List of Primary Outcome Measures from each Study Included in Sub-Group Analysis* | | | | | | | | | | |
| --- | --- | --- | --- | --- | --- | --- | --- | --- | --- | --- |
| Study ID | Primary outcome | Measure | Control group | intervention | | | control | | | *SMD* |
|  |  |  |  | *n* | *Mean* | *SD* | *n* | *Mean* | *SD* |  |
| Bierman (2015) | Emergent literacy | Letter-word identification scale; Woodcock-Johnson tests, Woodcock et al. 2001 | DI | 91 | 0.05 | 1.05 | 101 | -0.04 | 0.96 | 0.09 |
| Bleses, 2020 | Early numeracy skills | Mathematical checklist; developed by the researchers | DI | 538 | 28.2 | 9.9 | 578 | 26 | 9.5 | 0.23 |
| Borriello, (2018) | Spatial Language | Percentage of spatial language calculated, developed by researchers | FP | 19 | 8.6 | 3.8 | 22 | 5.7 | 2.6 | 0.89 |
| Casey (2008) | Spatial visualisation | Block design score; Wechsler intelligence scale, 4^th^ Ed., Wechsler, 2003 | DI | 16 | 14.5 | 8.49 | 10 | 13.6 | 6.19 | 0.12 |
| Cavanaugh (2017) | Early literacy skills | Dynamic Indicators of Basic Early Literacy; Skills, Kaminski & Good III, 1998 | DI | 21 | 11.7 | 6 | 20 | 7.2 | 8 | 0.63 |
| Christie (1983) | Receptive vocabulary | Peabody Picture Vocabulary Test; Dunn & Dunn, 2007 | DI | 8 | 80.2 | 18.1 | 9 | 77.4 | 19.7 | 0.14 |
| Cohrssen (2019) | Applied math problems | Woodcock-Johnson III; Mather &Woodcock, 2001 | DI | 22 | 11.3 | 4.83 | 38 | 12.3 | 5.08 | -0.21 |
| Coplan (2010) | Prosocial behaviour | Child Behaviour Scale; Ladd & Profilet, 1996 | DI | 11 | 2.26 | 0.44 | 11 | 2.3 | 0.36 | -0.10 |
| Conner (2013) | Pretend play | Play In Early Childhood Evaluation System (PIECES); Kelly-Vance & Ryalls, 2008 | FP | 5 | 32.12 | 20.29 | 5 | 30.64 | 29.88 | 0.05 |
| Dejonckheere (2016) | Exploratory play | Light-box task; developed by researchers | DI | 27 | 6.63 | 2.54 | 30 | 4.3 | 3.13 | 0.80 |
| Dempsey (2013) | Pretend play | Play In Early Childhood Evaluation System (PIECES); Kelly-Vance & Ryalls, 2008 | FP | 5 | 37.2 | 14.13 | 4 | 12 | 6.98 | 1.93 |
| Dickinson (2019) | Receptive vocabulary | Peabody Picture Vocabulary Test; Dunn & Dunn, 2007 | DI | 136 | 90.2 | 17.3 | 81 | 91.6 | 14 | -0.09 |
| Eason (2020) | Maths vocabulary | Total math talk calculated, developed by researchers | DI | 25 | 0.12 | 0.06 | 24 | 0.262 | 0.16 | -1.17 |
|  |  |  | FP | 25 | 0.12 | 0.06 | 23 | 0.04 | 0.03 | 1.69 |
| Ferrara (2011) | Spatial language | University of Chicago spatial language coding system; Cannon et al., 2007 | FP | 24 | 14 | 9 | 24 | 12 | 7 | 0.24 |
| Fisher (2011a) | Shape sorting | Shape sorting task (typical shapes); developed by researchers | DI | 14 | 0.86 | 0.24 | 14 | 0.8 | 0.27 | 0.23 |
| Fisher (2011b) | Shape sorting | Shape sorting task (typical shapes); developed by researchers | DI | 12 | 0.96 | 0.1 | 12 | 0.71 | 0.25 | 1.27 |
|  |  |  | FP | 12 | 0.96 | 0.1 | 12 | 0.53 | 0.19 | 2.73 |
| Goldstein (2018) | Emotion matching | Emotion matching; Bryant, 1982 | DI | 28 | 5.8 | 1.68 | 11 | 5.35 | 2.69 | 0.22 |
| Han (2010) | Expressive vocabulary | Picture Naming, Individual Growth and Development Indicators; Early Childhood Research Institute on Measuring Growth and Development, 2000 | DI | 24 | 7.37 | 5.03 | 25 | 3.88 | 4.95 | 0.69 |
| Jemutai (2019) | Visual perception | Visual Perception Aspects Test; Clutten, 2009 | DI | 18 | 3.78 | 6.03 | 20 | 1.95 | 5.56 | 0.31 |
| Kalkusch (2020) | Pretend play | Social Pretend Play Quality, developed by the researchers | FP | 30 | 2.65 | 0.49 | 24 | 2.17 | 0.52 | 0.94 |
| Lau (2005) | Social skills | Teacher Impression Scales; McConnell & Odom, 1993 | FP | 18 | 6.22 | 8.91 | 18 | 8.17 | 7.91 | -0.23 |
| Li (2016) | Prosocial behaviour | Play Observation Scale; Rubin, 2001 | DI | 8 | 9.62 | 3.42 | 8 | 1.63 | 1.92 | 2.72 |
| O'Connor (2011) | Total Language score | Preschool Language Scale -4; Zimmerman et al., 2002 | DI | 19 | 103 | 8.02 | 16 | 113 | 19.9 | -0.64 |
| Pearson (2008) | Social competence | The Social Competence and Behaviour Evaluation Scale-30, Lafreniere & Dumas, 1996 | FP | 16 | 48.6 | 6.31 | 16 | 47.9 | 7.18 | 0.09 |
| Sawyer (2019) | Spatial complexity | Drawing dimension, Consensual Assessment Technique; Amabile, 1982 | DI | 28 | 2.91 | 1.78 | 9 | 3.03 | 2.15 | -0.06 |
| Schmitt (2018) | Global executive function | Head Toes Knees Shoulders; Cameron Ponitz et al., 2008, 2009 | DI | 24 | 30.8 | 18.2 | 35 | 25 | 19 | 0.31 |
| Sinha (2012) | Behavioural regulation | Head Toes Knees Shoulders; Cameron Ponitz et al., 2008, 2009 | DI | 9 | 22.2 | 14.1 | 10 | 23.4 | 11.7 | -0.09 |
|  |  |  | FP | 10 | 22 | 13.4 | 10 | 20.9 | 13.8 | 0.08 |
| Thibodeau, 2016 | Inhibitory control | Day/Night Stroop task; Gerstadt et al. 1994 | DI | 34 | 11.5 | 4.72 | 31 | 12 | 5.52 | -0.10 |
| Toub (2018) | Expressive vocabulary | New Word Definition Test-Modified; Hadley et al., 2016 | DI | 83 | 0.59 | 0.48 | 82 | 0.64 | 0.51 | -0.10 |
|  |  |  | FP | 83 | 0.59 | 0.48 | 84 | 0.42 | 0.41 | 0.38 |
| van schijndel (2010) | Exploratory play | Exploratory play scale; developed by the researchers | FP | 19 | 2.52 | 0.42 | 9 | 2.37 | 0.3 | 0.38 |
| Note. Distinctions are made where studies compare guided play to both direct instruction and free play. Fisher (2011a) and Fisher (2011b) report on different participants. Fisher (2013) reports on the same participants as Fisher (2011b) with additional participants included but this data could not be separated and so Fisher (2013) is not included in analyses to ensure participants are not reported twice. | | | | | | | | | | |

|  | **Table S4.** *Additional Characteristics of Included Studies* | | | | | | | | | | | |
| --- | --- | --- | --- | --- | --- | --- | --- | --- | --- | --- | --- | --- |
| *Author (Year)* | | Study Design^a^ | Child's Age (Years) | Intervention Location^b^ | Adult Present | Country | Total Participants | Number of Exposures | Main Outcome(s) | Intervention Content  (Guidance methods identified) | Type of Play | Control^c^ |
| Bierman (2015) | | RE | 3-6 | Home | Parent | USA | 100 to 199 | 6 to 20 | Literacy and social skills | Parents provided with role play resources (e.g., Chef) and instructed to play with child and initiate conversations. Parents trained on importance of play and given instructions to support guiding child. (Open-ended questions, model, co-play) | Pretend | DI |
| Bleses (2020) | | RE | 1-3 | ECC | Teacher | Denmark | 200+ | 21+ | Language and maths | Teachers trained in using responsive and differential strategies in teaching and supported in implementing lesson plans which used playful activities and ‘exploratory zones’ to promote children’s engagement and support quality interactions. (Open-ended questions, adjust to child’s level, co-play) | Unclear | DI |
| Borriello (2018) | | QE | 3-6 | Lab | Parent | USA | 0 to 49 | 1 to 5** | Maths/ spatial language | Mother-child dyads engaged in free play with Lego. Mothers were given examples of guidance and encouraged to use guidance and spatial language throughout. (Model, co-play) | Object | FP |
| Bulunuz (2013) | | QE | 3-6 | Classroom | Teacher | Turkey | 0 to 49 | 21+ | Science concepts | Curriculum approach to learning about scientific concept through play; adult-initiated playful activities set up in the class relating to science topics (e.g., sink and float). (Open-ended questions, modelling) | Exploratory | DI |
| Casey (2008) | | QE | 3-6, 6-8 | Classroom | Teacher | USA | 100 to 199 | 6 to 20 | Maths-spatial skills | Children are first exposed to a relevant story and then challenged to build a structure from the story using blocks e.g., Queens’s castle. Complexity of building required increases with each exposure to intervention. (Open-ended questions, sets challenge) | Object | DI |
| Cavanaugh (2017) | | QE | 3-6 | Classroom | Teacher | USA | 0 to 49 | 6 to 20 | Literacy/ language | Following a teachers’ example, children are provided with pots of phonics-related items and encouraged to create their own game e.g., Sorting game, goodie vs baddie stories. (Model, set challenge, hint/prompt) | Pretend | DI |
| Christie (1983) | | QE | 3-6 | Classroom | Researcher | USA | 0 to 49 | 6 to 20 | Literacy – vocabulary, play and creativity | Children engaged in sociodramatic play within various themes and props (e.g., hospital, cooking). An adult encouraged fantasy play using various techniques. (Model, hint/prompt, suggestion, co-play, adjust to child’s level) | Pretend | DI |
| Cohrssen (2019) | | QE | 3-6 | Classroom | Teacher | Australia | 50 to 99 | 21+ | Maths skills | Play-based maths curriculum; set math games lead by the teacher e.g., Sorting and counting with pebbles. (Hint/prompt, open-ended questions) | Exploratory and object | DI |
| Conner (2013) | | QE | 1-3 | ECC | Researcher | USA | 0 to 49 | 6 to 20 | Literacy/ language | Small groups of children were read a book and then introduced to related play resources. Children could play freely while the experimenter facilitated play through modelling behaviours.  (Model) | Exploratory and pretend | FP |
| Coplan (2010) | | RE | 3-6 | ECC | Researcher * | Canada | 0 to 49 | 6 to 20 | Social skills | Following circle time introductions, a small group of children engaged in free play with resources while an adult supported and facilitated social interactions as they played. (Model, hint/prompt, open-ended questions) | Unclear | DI |
| Dejonckheere (2016) | | RE | 3-6 | Classroom | Teacher | USA | 50 to 99 | Unclear | Exploratory play | Teacher provided materials for science-based exploratory play and demonstrated their uses (e.g., magnets, objects that float/sink). Children were free to engage in play with the materials and the teacher provided guidance to focus the children’s exploration. (Model, suggestion, open-ended questions, set challenge) | Exploratory | DI |
| Dempsey (2013) | | RE | 1-3, 3-6 | Home, ECC | Parent | USA | 0 to 49 | 21+ | Play (pretend and exploratory) | Parents were trained on guidance strategies (e.g., modelling, prompts, add-ons) to implement during parent-child play. Parents were encouraged to implement daily for 6 weeks when their child played. (Model, hint/prompt, co-play) | Exploratory and pretend | FP |
| Dickinson (2019) | | RE | 3-6 | Classroom | Teacher | USA | 200+ | 21+ | Literacy- vocabulary | Intervention combined book reading and play with related resources to improve language learning of target words. Following book reading, teachers first re-enacted the story with props before facilitating language use of children as they played more freely with props. (Open-ended questions, hint/prompt, co-play) | Pretend | DI |
| Eason (2020) | | RE | 3-6 | Lab | Parent | USA | 50 to 99 | 1 to 5** | Maths vocabulary | Parent-child dyads were provided with wooden food items which could be partitioned and followed a story book which prompted children to share and explore the food items to learn about fractions through a playful context. (Open-ended questions, hint/prompt, co-play) | Pretend | DI, FP |
| Ferrara (2011) | | RE | 3-6 | Unclear | Parent | USA | 0 to 49 | 1 to 5** | Maths-spatial language | Children and parents provided with images of structures to build with blocks (e.g. vehicles). Parents are encouraged to support the child as they build. (Set challenge, co-play) | Object | FP |
| Fisher (2011a) | | RE | 3-6 | Lab | Researcher | USA | 0 to 49 | 1 to 5** | Maths-shape knowledge | Researchers encourage children to explore laminated shapes and use questions and prompts to support children in identifying shape features. (Model, hint/prompt, open-ended questions) | Exploratory and object | DI |
| Fisher (2011b) | | RE | 3-6 | Classroom | Researcher | USA | 0 to 49 | 1 to 5** | Maths-shape knowledge | As above but with added playful element of children pretending to be detectives to discover the shapes features. Children then created novel shapes from sticks with scaffolded support from adults. (Hint/prompt, open-ended questions, adjust to child’s level) | Exploratory and object | DI, FP |
| Fisher (2013) | | QE | 3-6 | Classroom & lab | Researcher | USA | 50 to 99 | 1 to 5** | Maths-shape knowledge | As above. (Hint/prompt, open-ended questions) | Exploratory and object | DI, FP |
| Gmitrova (2013) | | RE | 3-6 | Classroom | Teacher | Slovak Republic | 200+ | 1 to 5** | Play | The teacher initiated a pretend play session according to a pre-defined scenario with relevant toys (e.g. doctor). The teacher modelled roles for children and joined in the play. (Model, co-play) | Pretend | FP |
| Goldstein (2018) | | RE | 3-6 | Classroom | Researcher | USA | 50 to 99 | 21+ | Emotional control | 2 conditions involving guided play: 1) Children were asked to build set structures from blocks (e.g. Giraffe, trees) while an adult facilitated this play. 2) children were encouraged to take part in role play scenarios with adult support (e.g. chef).  (Model, hint/prompt) | Object and pretend | DI |
| Golomb (1977) | | QE | 3-6 | Unclear | Researcher | USA | 0 to 49 | 1 to 5 | Conservation judgement | With adult support, children engaged in pretense play activities involving role play (e.g. playdough food picnic, driving to the mountains). Adults then challenged the pretense. (Hint/prompt, open-ended questions, co-play) | Pretend | DI |
| Han (2010) | | RE | 3-6 | Classroom | Researcher * | USA | 0 to 49 | 21+ | Literacy- vocabulary | EIVP+play; children were engaged in a joint book reading activity followed by a play episode where target words were acted out by children using props and adult guidance (e.g. baking).  (Model, co-play) | Pretend | DI |
| Jemutai (2019) | | QE | 3-6, 6-8 | Classroom | Teacher | South Africa, Kenya | 50 to 99 | 21+ | Visuospatial abilities | 6 bricks intervention: teachers were trained in using guidance techniques while children engaged in 6 brick activities. Teachers would demonstrate activities and then support children as they explored further. (Model) | Object | DI |
| Kalkusch (2020) | | RE | 3-6 | ECC | Researcher | Switzerland | 50 to 99 | 6 to 20 | Pretend play | An adult ‘play tutor’ engaged children in pretend play involving various themes with props/toys (e.g. firefighters). They modelled play actions and used other strategies to actively support children’s play. They were flexible and took children’s ideas and needs into account. (Model, hint/prompt, adjust to child’s level) | Pretend | FP |
| Lau (2005) | | QE | 3-6 | Classroom | Teacher | USA | 0 to 49 | 21+ | Social interactions | Teachers-facilitated social interactions though prompts and cues while pairs of children engaged with ‘Elmo Arts’ computer game. (Hint/prompt) | Unclear | FP |
| Li (2016) | | RE | 3-6 | Classroom | Other | China | 0 to 49 | 6 to 20 | Social skills | Following circle time introductions, a small group of children engaged in free play with resources while an adult supported and facilitated social interactions as they played.  (model, hint/prompt, open-ended questions) | Unclear | DI |
| Morris (2018) | | RE | 3-6 | Classroom | Teacher | Australia | 200+ | 6 to 20 | Knowledge of wellbeing/ sustainability | Teachers attended ‘learning sessions’ on implementing play-based learning with an emphasis on scaffolding. Open-ended, modelled, and purposefully framed play were then implemented in the classroom for children to engage with. (Model, set challenge, co-play) | Unclear | DI |
| O’Connor (2011) | | QE | 3-6, 6-8 | Classroom | Other | Australia | 0 to 49 | 21+ | Play, language and social skills | ‘Learn to play’ intervention; child-led play occurred in different play stations (doll play, transport play, construction, and home corner) while the adult facilitated play and social interactions. (Model, co-play) | Pretend | DI |
| Palma (2014) | | RE | 3-6 | Classroom | Teacher | Portugal | 50 to 99 | 21+ | Gross motor skills | PE teachers set up activities for children to engage with to develop physical motor skills. During play, adults encouraged children’s exploration through modelling and reinforcing activity. (Model, adjust to child’s level) | Physical | DI, FP |
| Pearson (2008) | | RE | 3-6 | Classroom | Researcher | USA | 0 to 49 | 1 to 5 | Problem solving and emotions | As part of a cognitive behavioural intervention, children were presented with common problems using dolls. The children then continued the story using doll play and with support from adults. Children also had opportunity to make up their own stories. (Model, hint-prompt, co-play) | Pretend | FP |
| Pellegrini (1980) | | QE | 3-6 | ECC | Researcher | USA | 0 to 49 | 1 to 5** | Language-associative fluency | Children were given 2 objects to play with (thread spool, pipe cleaner, clothespin, bottle cork). Open-ended questions were used by adults to support children’s exploration of objects. (Open-ended questions) | Exploratory | DI, FP |
| Sawyer (2019) | | RE | 3-6 | Classroom | Researcher | USA | 50 to 99 | 21+ | Drawing development | 2 conditions involving guided play: 1) Children were asked to build set structures from blocks (e.g. Giraffe, trees) while an adult facilitated this play. 2) children were encouraged to take part in role play scenarios with adult support (e.g. chef). (Model, hint/prompt) | Object and pretend | DI |
| Schmitt (2018) | | RE | 3-6 | Classroom | Researcher | USA | 50 to 99 | 6 to 20 | Maths and executive function | In small groups, children were given block building tasks to achieve. Adults scaffolded learning by increasing the complexity of builds with each exposure. (Hint/prompt, set challenge) | Object | DI |
| Sinha (2012) | | QE | 3-6 | Classroom | Researcher | USA | 50 to 99 | 1 to 5 | Self-regulation | Small groups of children were presented with an array of play materials (masks, puppets, household items) and encouraged to play freely while the adult supported interactions in the group, encouraging imagination and interactive dialogue. (Model, co-play) | Pretend | DI, FP |
| Smith (1978) | | QE | 3-6 | Private residence | Unclear | UK | 0 to 49 | 6 to 20 | Social participation and play | An adult ‘play tutor’ initiated and maintained children’s fantasy play by providing a theme (e.g. farm) and materials, and encouraged children to join in. (Suggestion) | Pretend | DI |
| Smith (1981) | | QE | 3-6 | ECC | Researcher * | UK | 50 to 99 | 21+ | Social participation, cognitive ability and play | An adult ‘play tutor’ encouraged children’s fantasy/sociodramatic play. They followed the child’s lead but offered ideas and information. (Suggestion, adjust to child’s level) | Pretend | DI |
| Thibodeau (2016) | | RE | 3-6 | Classroom | Researcher | USA | 50 to 99 | 21+ | Executive function | Small groups of children were supported to develop play scripts using their imaginations and guided while they acted out the plays using scaffolding when needed. (Hint/prompt, adjust to child’s level, open-ended questions) | Pretend | DI |
| Toub (2018) | | RE | 3-6 | Classroom | Researcher | USA | 200+ | 6 to 20 | Literacy - vocabulary | Following book reading, children were given props relating to the story to use however they wished. An adult joined in the child’s play and used guidance methods to expose them to target words at naturally occurring moments. (Open-ended questions, co-play, adjust to child’s level) | Pretend | DI, FP |
| van schijndel (2010) | | QE | 1-3 | ECC | Teacher | Netherlands | 0 to 49 | 6 to 20 | Exploratory play | Small groups of children interacted with science-based resources and an adult in a sandpit. Teacher encouraged participation and exploration through questions and modelling as children explored ‘sorting’ and ‘speed’-related props and themes. (Hint/prompt, open-ended questions, model, co-play) | Exploratory | FP |
|  | Note. ^a^ RE= randomised experimental design, QE = quasi-experimental design. ^b^ ECC = Early childhood care and education setting.  ^c^ DI = direct instruction/treatment as usual, FP = free play.  * Outside member e.g. tutor, group leader, but trained by research team ** Single exposure to intervention | | | | | | | | | | | |

**Figure S1.** *Risk of Bias Summary.* The Cochrane Risk of Bias Tool (Higgins & Green, 2011) was used to assess the risk of bias across studies (*N* = 39)

| Random sequence generation | |  |  | \| 3% \| 25% \| 72% \| \| --- \| --- \| --- \| | | |
| --- | --- | --- | --- | --- | --- | --- | --- | --- | --- |
| Allocation concealment | |  |  | \|  \| 97% \| 3% \| \| --- \| --- \| --- \| | | |
| Blinding of participants and personnel | |  |  | \| 3% \| 20% \| 77% \| \| --- \| --- \| --- \| | | |
| Blinding of outcome assessment | |  |  | \| 33% \| 29% \| 38% \| \| --- \| --- \| --- \| | | |
| Incomplete outcome data | |  |  | \| 72% \| 20% \| 8% \| \| --- \| --- \| --- \| | | |
| Selective reporting | |  |  | \| 3% \| 94% \| 3% \| \| --- \| --- \| --- \| | | |
| Other bias | |  |  | \| 15% \| 6% \| 79% \| \| --- \| --- \| --- \| | | |
|  |  | | | | \|  \| Low risk: \|  \| Unclear risk: \|  \| High risk: \|  \|  \| \| --- \| --- \| --- \| --- \| --- \| --- \| --- \| --- \| |  |

**Figure S2.** *Funnel Plot of All Quantitative Studies (n = 30)*


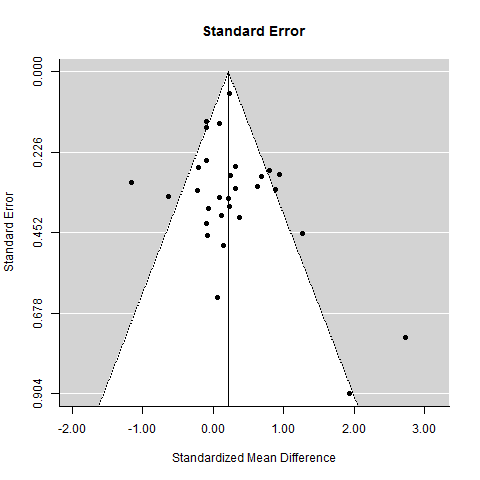


**Figure S3.** *Forest Plot of Meta-Analysis Result for Early Literacy Skills with Direct Instruction as Comparison Group*


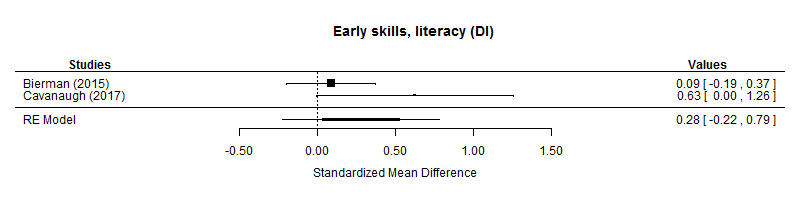


Bierman (2015), Letter-word identification scale ^1^

Cavanaugh (2017), DIBELS ^2^

**Figure S4.** *Forest Plot of Meta-Analysis Result for Expressive Vocabulary with Direct Instruction as Comparison Group*


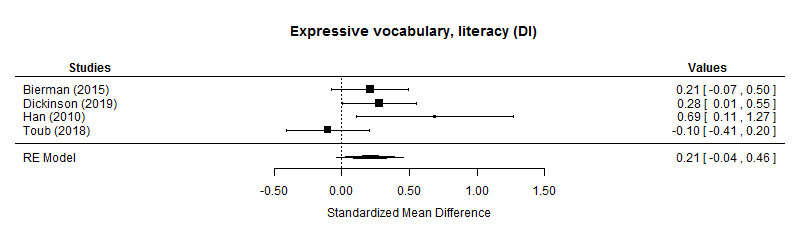


Bierman (2015), One-word picture vocabulary test ^3^

Dickinson (2019), New word definition test-modified ^4^

Han (2010), Picture naming, ECRI ^5^

Toub (2018), New word definition test-modified ^4^


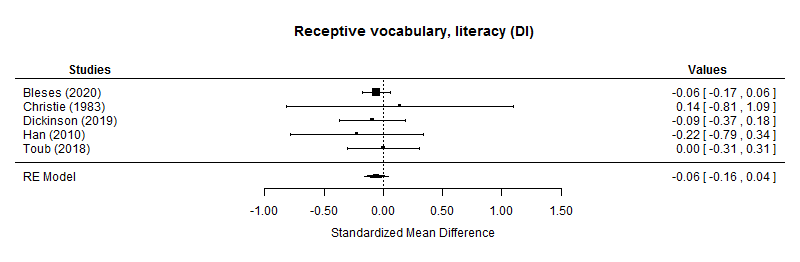
**Figure S5.** *Forest Plot of Meta-Analysis Result for Receptive Vocabulary with Direct Instruction as Comparison Group*

Bleses (2020), CDI-educator*

Christie (1983), PPVT ^6^

Dickinson (2019), PPVT ^6^

Han (2010), PPVT ^6^

Toub (2018), PPVT ^6^

**Figure S6.** *Forest Plot of Meta-Analysis Result for Early Maths Skills with Direct Instruction as Comparison Group*


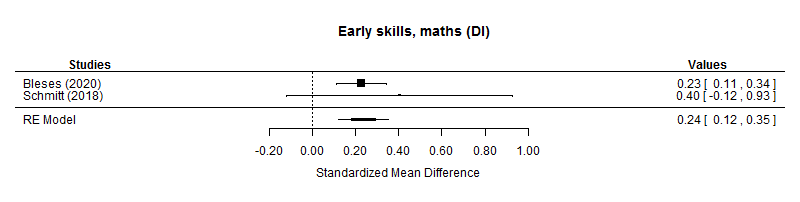


Bleses (2020), Mathematical checklist*

Schmitt (2018), Preschool early numeracy skills ^7^

**Figure S7.** *Forest Plot of Meta-Analysis Result for Shape Knowledge with Direct Instruction as Comparison Group*


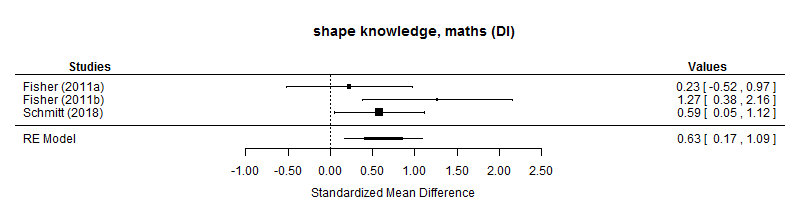


Fisher (2011a), Shape sorting task (typical shapes)*

Fisher (2011b), Shape sorting task (typical shapes)*

Schmitt (2018), Shape recognition task ^8^

**Figure S8.** *Forest Plot of Meta-Analysis Result for Spatial and Mathematic Vocabulary with Direct Instruction as Comparison Group*


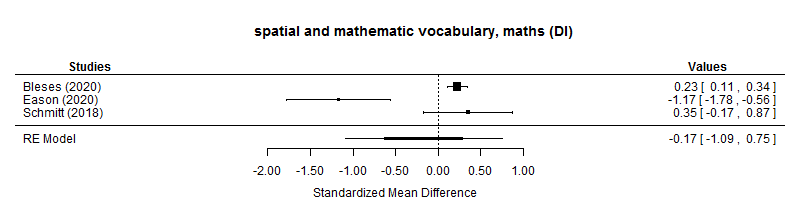


Bleses (2020), Mathematical checklist*

Eason (2020), Total math talk*

Schmitt (2018), Mathematical language*

**Figure S9.** *Forest Plot of Meta-Analysis Result for Spatial and Mathematic Vocabulary with Free Play as Comparison Group*


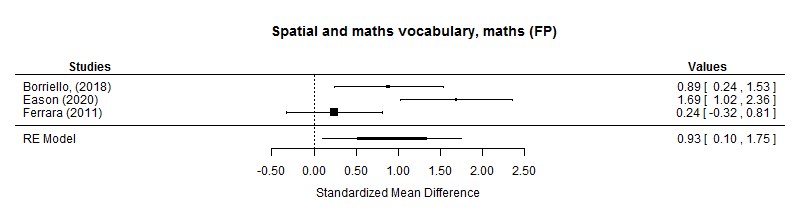


Borriello (2018), Percentage of spatial talk*

Eason (2020), Total math talk*

Ferrara (2011), Spatial language*

**Figure S10.** *Forest Plot of Meta-Analysis Result for Task Switching with Direct Instruction as Comparison Group*


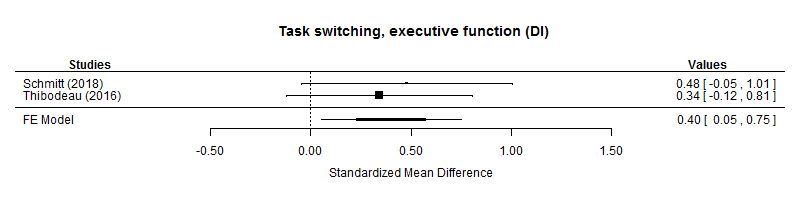


Schmitt (2018), Card sort task ^9^

Thibodeau (2016), Card sort task ^9^

**Figure S11.** *Forest Plot of Meta-Analysis Result for Behaviour Regulation with Direct Instruction as Comparison Group*


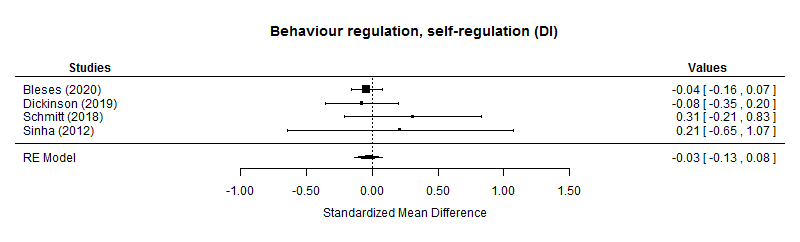


Bleses (2020), Self-regulation and cooperation index ^10^

Dickinson (2019), Peg tapping ^11^

Schmitt (2018), HTKS ^12^

Sinha (2012), HTKS ^12^

**Figure S12.** *Forest Plot of Meta-Analysis Result for Inhibitory Control with Direct Instruction as Comparison Group*


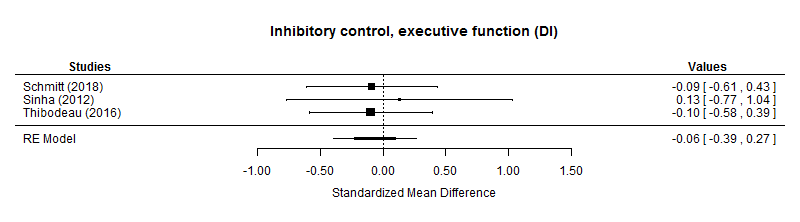


Schmitt (2018), Sun/moon Stroop task ^13^

Sinha (2012), Chimeric animal Stroop task ^14^

Thibodeau (2016), Day/night Stroop task ^15^

**Figure S13.** *Forest Plot of Meta-Analysis Result for Prosocial Behaviour with Direct Instruction as Comparison Group*


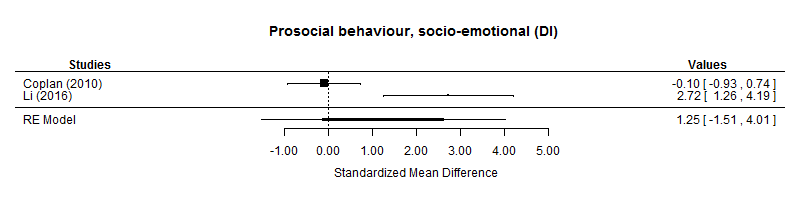


Coplan (2010), Child behaviour scale ^16^

Li (2016), Play observation scale ^17^

**Figure S14.** *Forest Plot of Meta-Analysis Result for Social Competence with Direct Instruction as Comparison Group*

**
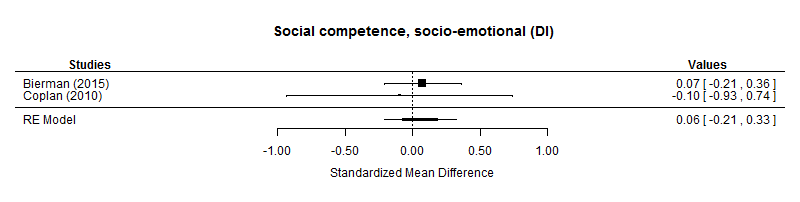
**

Bierman (2015), Social competence scale ^18^ *

Coplan (2010), Play observation scale ^17^

**Figure S15.** *Forest Plot of Subgroup Analysis for 1 to 5 Intervention Exposures*


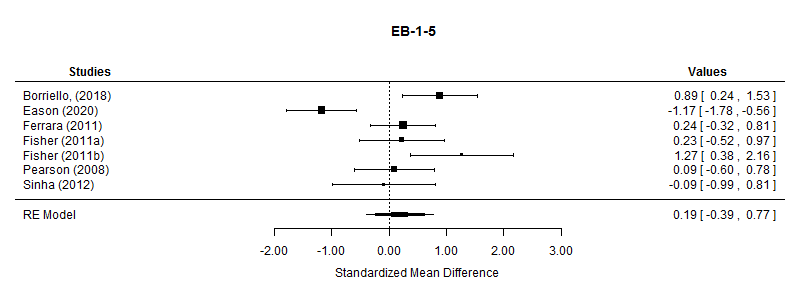
**Subgroup – Exposures (1 to 5)**

**Figure S16.** *Forest Plot of Subgroup Analysis for 6 to 20 Intervention Exposures*


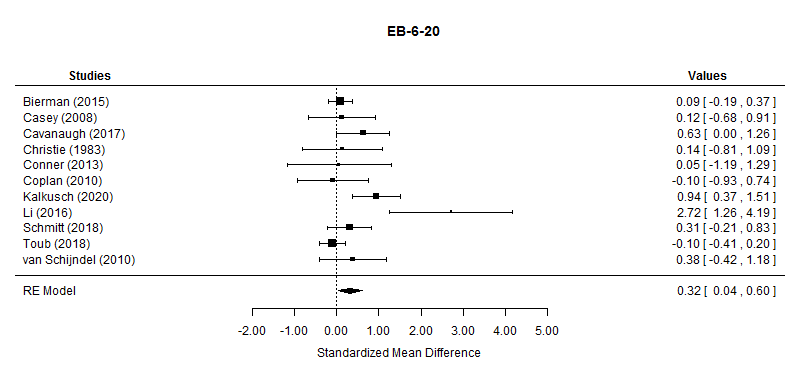
**Subgroup – Exposures (6 to 20)**

**Figure S17.** *Forest Plot of Subgroup Analysis for 21 or More Intervention Exposures*


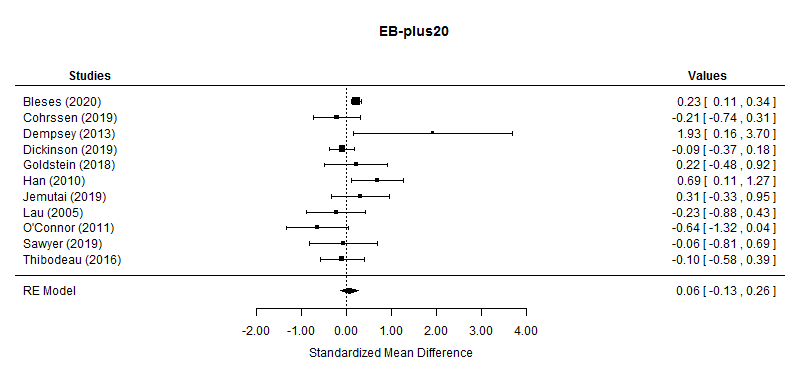
**Subgroup – Exposures (21+)**

**Figure S18.** *Forest Plot of Subgroup Analysis for Parent as Adult Present*


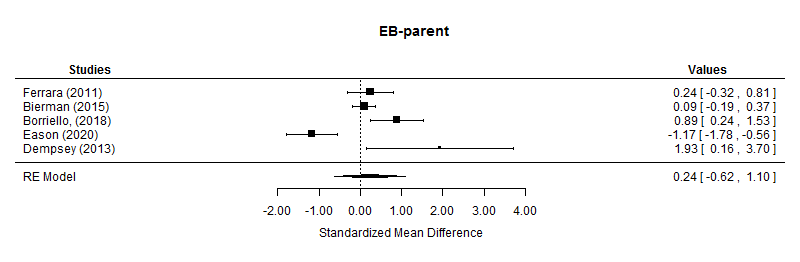
**Subgroup – Adult (parent)**

**Figure S19.** *Forest Plot of Subgroup Analysis for Teacher as Adult Present*


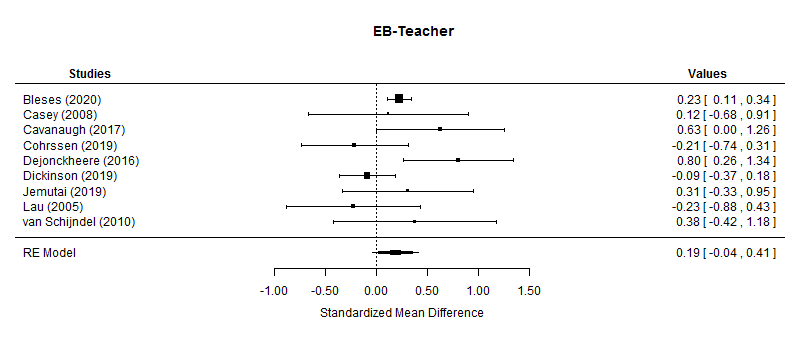
**Subgroup – Adult (teacher)**

**Figure S20.** *Forest Plot of Subgroup Analysis for Researchers as Adult Present*

**Subgroup – Adult (researcher)**


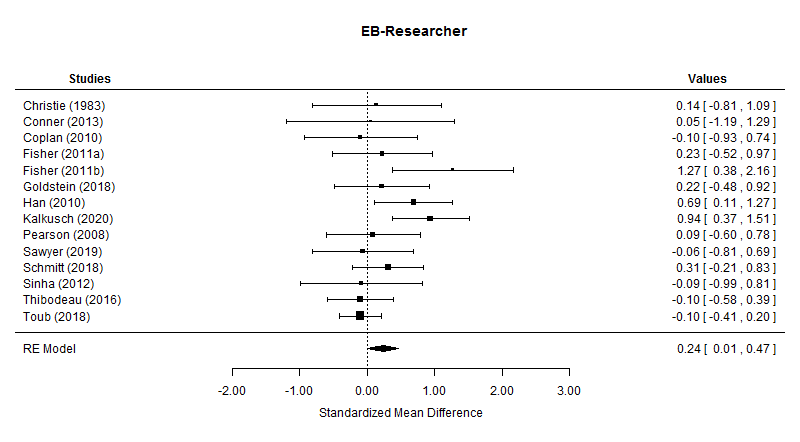


**Figure S21.** *Forest Plot of Subgroup Analysis for Less Than 50 Participants*


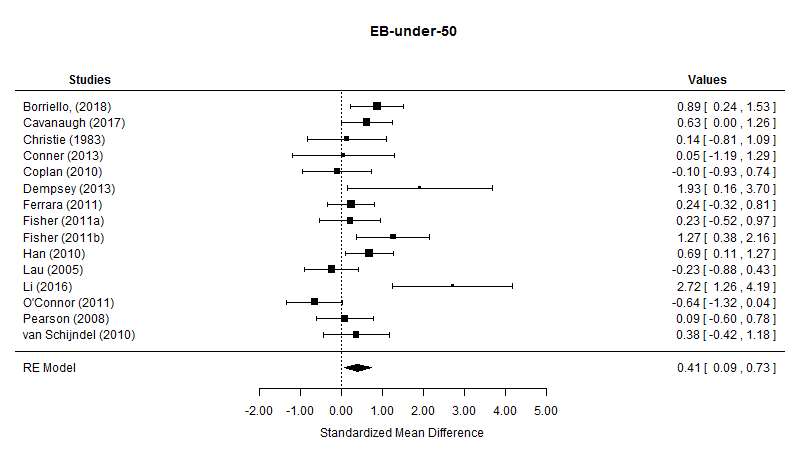
**Subgroup – Participant number (under 50)**

**Figure S22.** *Forest Plot of Subgroup Analysis for Over 50 Participants*


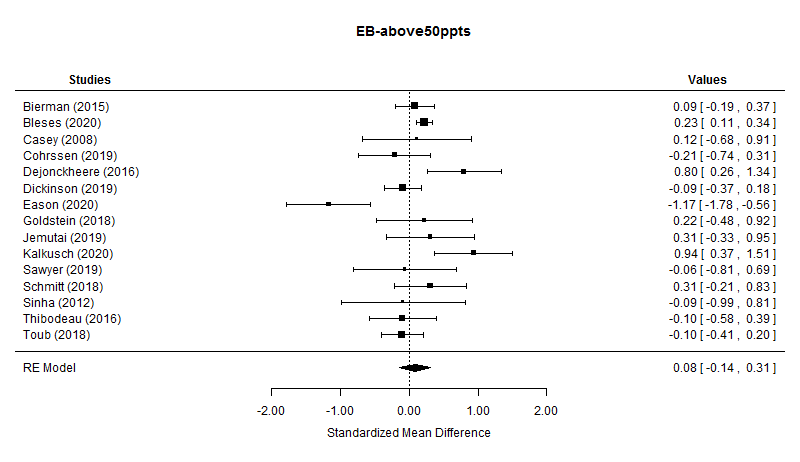
**Subgroup – Participant number (over 50)**

**Figure S23.** *Forest Plot of Subgroup Analysis for Studies with Free Play as Comparison Group*


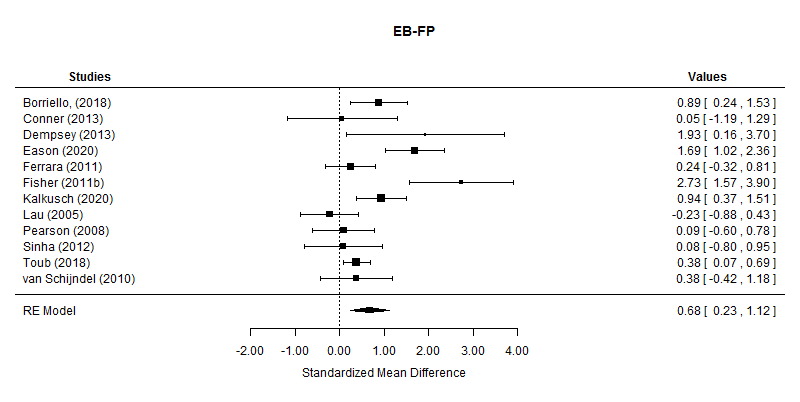
**Subgroup – Comparison (FP)**

**Figure S24.** *Forest Plot of Subgroup Analysis for Studies with Direct Instruction as Comparison Group*


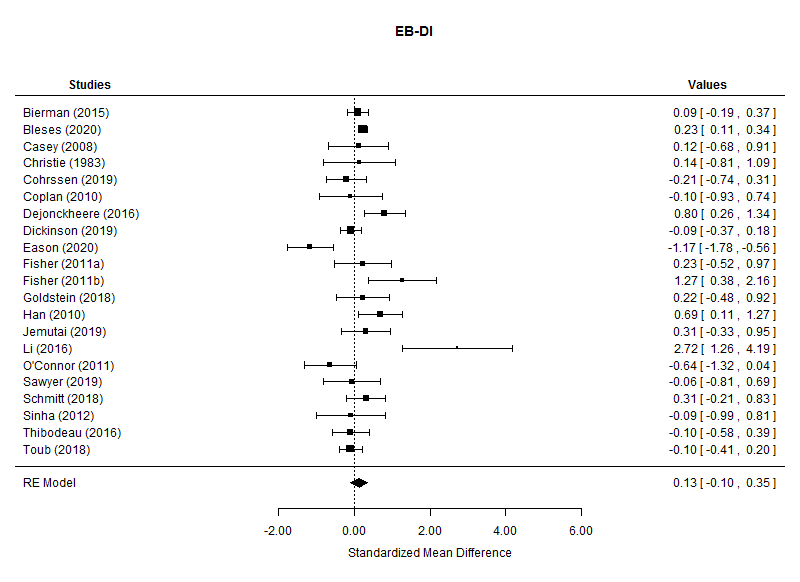
**Subgroup – Comparison (DI)**

**Figure S25.** *Forest Plot of Subgroup Analysis for Studies with RCT Study Design*


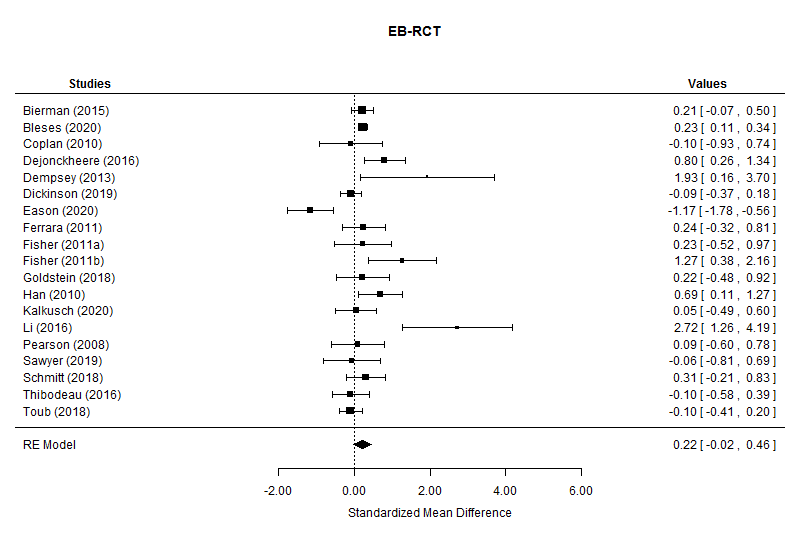
**Subgroup – Design (RCT)**

**Figure S26.** *Forest Plot of Subgroup Analysis for Studies with Quasi-Experimental Study Design*


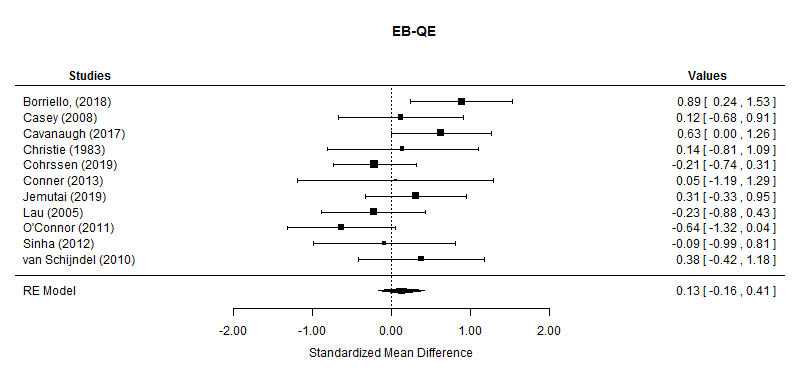
**Subgroup – Design (quasi)**

**Appendix S8. References for outcome measures in Table S3 and forest plots**

Amabile, T. M. (1982). A consensual assessment technique. Journal of Personality and Social Psychology, 43, 997–1013.

Archibald, S. J., & Kerns, K. A. (1999). Identification and description of new tests of executive functioning in children. Child Neuropsychology, 5, 115–129.

Brownell, R. (2000). Expressive One-Word Picture Vocabulary Test manual. Novato, CA: Academic Therapy.

Bryant, B.K. (1982). An index of empathy for children and adolescents. Child Development, 53, 413–425.

Cameron Ponitz, C., McClelland, M. M., Jewkes, A. M., Connor, C. M. D., Farris, C. L., & Morrison, F. J. (2008). Touch your toes! Developing a direct measure of behavioral regulation in early childhood. Early Childhood Research Quarterly, 23(2), 141–158.

Cameron Ponitz, C., McClelland, M. M., Matthews, J. S., & Morrison, F. J. (2009). A Structured Observation of Behavioral Self-Regulation and Its Contribution to Kindergarten Outcomes. Developmental Psychology, 45(3), 605–619.

Cannon, J., Levine, S., & Huttenlocher, J. (2007). A system for analyzing children and caregivers’ language about space in structured and unstructured contexts. Spatial Intelligence and Learning Center (SILC) technical report.

Clements, D. H., Swaminathan, S., Hannibal, M. A. Z., & Sarama, J. (1999). Young children’s concepts of shape. Journal for Research in Mathematics Education, 30, 192–212.

Clutten, S.C., 2009, ‘The development of a visual perception test for learners in the foundation phase’, Unpublished Master’s Dissertation, University of South Africa, Pretoria.

Conduct Problems Prevention Research Group. (1995). Social health profile. Retrieved from http://www.fasttrack project.org/

Diamond, A., & Taylor, C. (1996). Development of an aspect of executive control: Development of the abilities to remember what I said and to “Do as I say, not as I do”. Developmental Psychobiology, 29(4), 315–334.

Dunn, L. M., & Dunn, D. M. (2007). Peabody picture vocabulary test, fourth edition. Minneapolis, MN: NCS Pearson.

Early Childhood Research Institute on Measuring Growth and Development. (2000). Individual growth and development indicators for preschool children (IGDI).

Gerstadt, C. L., Hong, Y. J., & Diamond, A. (1994). The relationship between cognition and action: performance of children 3 1 2-7 years old on a Stroop- like day-night test. Cognition, 53(2), 129–153.

Hadley, E. B., Dickinson, D. K., Hirsh-Pasek, K., Golinkoff, R. M., & Nesbitt, K. T. (2016). Examining the acquisition of vocabulary knowledge depth among preschool students. Reading Research Quarterly, 51(2), 181–198.

Kaminski, R. A., & Good III, R. H. (1998). Assessing early literacy skills in a Problem-Solving model: Dynamic Indicators of Basic Early Literacy Skills. In Advanced applications of Curriculum-Based Measurement. (pp. 113–142). The Guilford Press.

Kelly-Vance, L., Ryalls, B. O. (2008). Best practices in play assessment and intervention. In Th. J. Grimes (Ed.), Best practices in school psychology (5th ed., p. 549–560). Bethesda, MD: National Association of School Psychologists.

Ladd, G. W., & Profilet, S. M. (1996). The Child Behavior Scale: A teacher-report measure young children’s aggressive, withdrawn, and prosocial behaviors. Developmental Psychology, 32, 1008–1024.

LaFreniere, P.J., & Dumas, J.E. (1996). Social competence and behavior evaluation in children ages 3 to 6 years: The short form (SCBE-30). Psychological Assessment, 8, 369-377.

McConnell, S. R., & Odom, S. L. (1993). Teacher impressions scales (TIS). In S. R. McConnell & S. L. Odom (Eds.), Play time/social time: Organizing your classroom to build interaction skills (pp. 12–13). Tucson, AZ: Communication Skill Builder.

Purpura, D. J., Reid, E. E., Eiland, M. D., & Baroody, A. J. (2015). Using a brief preschool early numeracy skills screener to identify young children with mathematics difficulties. School Psychology Review, 44(1), 41–59.

Rubin, K. H. (2001). The Play Observation Scale (POS). University of Maryland.

Squires, J. (2014). Social–emotional assessment/evaluation measure (seam). Paul H. Brookes Publishing.

Wechsler, D. (2003). Wechsler intelligence scale for children–Fourth Edition (WISC-IV). San Antonio, TX: The Psychological Corporation.

Woodcock, R. W., McGrew, K. S., & Mather, N. M. (2001). Woodcock–Johnson III: Tests of cognitive abilities. Itasca, IL: Riverside.

Wright, I., Waterman, M., Prescott, H., & Murdoch-Eaton, D. (2003). A new Stroop-like measure of inhibitory function. Journal of Child Psychology and Psychiatry and Allied Disciplines, 44, 561–575.

Zelazo, P. D. (2006). The Dimensional Change Card Sort (DCCS): A method of assessing executive function in children. Nature Protocols, 1(1), 297–301.

Zimmerman, I. L., Steiner, V. G., & Pond, R. E. (2002). Preschool Language Scale: Examiner’s manual (4th ed.). San Antonio, TX: Pearson.

**Table S5.** Details of the supplementary literature search strategy based on search terms suggested during peer review. The search was conducted via electronic databases on 16/02/2021.

|  | Database(s) | | | | | | | | | | | |
| --- | --- | --- | --- | --- | --- | --- | --- | --- | --- | --- | --- | --- |
|  | ERIC | | | BEI | | | PsychInfo & PsychArticles | | | Child dev. & adolescent studies, Scopus, & WoS | | |
|  | Term | Free | MeSH | Term | Free | MeSH | Term | Free | MeSH | Term | Free | MeSH |
| 1 | DE “inquiry” |  | x | DE “process-orientated guided inquiry learning” |  | x | DE “discovery teaching method” |  | x | “discovery based learning” | x |  |
| 2 | DE “active learning” |  | x | DE “learning by discovery” |  | x | DE “experiential learning” |  | x | “discovery learning” | x |  |
| 3 | DE “experiential learning” |  | x | DE “experiential learning” |  | x | “guided discovery” | x |  | “inquiry based learning” | x |  |
| 4 | DE “discovery learning” |  | x | DE “active learning” |  | x | “guided participation” | x |  | “inquiry learning” | x |  |
| 5 | “guided discovery” | x |  | DE “inquiry-based learning” |  | x | “inquiry learning” | x |  | “experiential learning” | x |  |
| 6 | “guided participation” | x |  | “guided discovery” | x |  | “discovery learning” | x |  | “guided discovery” | x |  |
| 7 | OR/1-6 | | | “guided participation” | x |  | OR/1-6 | | | “guided participation” | x |  |
| 8 | DE “play” |  | x | OR/ 1-7 | | | play | x |  | OR/ 1-7 | | |
| 9 | 7 AND 8 | | | DE “play” |  | x | 7 AND 8 | | | play | x |  |
| 10 | “play tutor*” | x |  | 8 AND 9 | | | “play tutor” | x |  | 8 AND 9 | | |
| 11 | 9 OR 10 | | | “play tutor*” | x |  | “play tutoring” | x |  | “play tutor” | x |  |
| 12 | 11 AND [RD terms**] | | | 10 AND 11 | | | OR/ 10-11 | | | “play tutoring” | x |  |
| 13 |  | | | 12 AND [RD terms*] | | | 9 OR 12 | | | 11 OR 12 | | |
| 14 |  |  |  |  | | | 13 AND [RD terms*] | | | 10 OR 14 | | |
| 15 |  |  |  |  |  |  |  | | | 14 AND [RD terms*] | | |

*Note.* RD = research design.

** Database-specific (see Table S1 Continued)

**Table S6.** Summary of additional sample characteristics and context.

| *Author (Year)* | *Study country* | *Gender (child sample)* | *Race/ethnicity (child sample)* |
| --- | --- | --- | --- |
| Bierman (2015) | USA | 56% male, 44% female | 55% Caucasian, 26% African American 19% Latino |
| Bleses  (2020) | Denmark | Intervention: 48% male, 52% female  Control: 49% male, 51% female | Intervention: 9% non-Western origin  Control: 12% non-Western origin |
| Borriello (2018) | USA | Intervention: 58% male, 42% female  Control: 50% male, 50% female | 85% Caucasian, 7% Asian American, 5% Hispanic, 2% African American |
| Bulunuz  (2013) | Turkey | Intervention: 42% male, 58% female  Control: 64% male, 36% female | Unclear |
| Casey  (2008) | USA | Male & female sample (% unclear) | School A: 60% African American, 29% Hispanic, 8% Caucasian, 2% Asian, 1% Native American  School B: 14% African American, 29% Hispanic, 48% Caucasian, 9% Asian, 0% Native American |
| Cavanaugh (2017) | USA | 46% male, 54% female | 66% Caucasian, 27% African American, 7% Hispanic |
| Christie  (1983) | USA | Unclear | Limited data provided; most participants were Caucasian, plus some who were Black, Mexican American, and Native American |
| Cohrssen (2019) | Australia | Intervention: 18% male, 82% female  Control: 58% male, 42% female | Based on participating *school* population rather than sample.  Intervention: 52% indigenous student enrolment  Control: 8% indigenous student enrolment |
| Conner  (2013) | USA | Intervention: 80% male, 20% female  Control: 80% male, 20% female | 100% Caucasian |
| Coplan  (2010) | Canada | 50% male, 50% female | 72% Caucasian, 9% Asian, 5% Black, 14% other/unclear |
| Dejonckheere (2016) | USA | 54% male, 46% female | Unclear |
| Dempsey (2013) | USA | Intervention: 60% male, 40% female  Control: 25% male, 75% female | Intervention: 100% Caucasian  Control: 50% Caucasian, 25% Native American, 25% Hispanic |
| Dickinson (2019) | USA | 48% male, 52% female | 42% African American, 32% Hispanic/Latino, 15% European American, 9% Multiracial/Other |
| Eason  (2020) | USA | 42% male, 58% female | 60% Caucasian, 29% mixed race, 7% Black, 4% Asian |
| Ferrara  (2011) | USA | Male & female sample (% unclear) | Unclear (described as predominantly Caucasian) |
| Fisher  (2011a) | USA | Male & female sample (% unclear) | Unclear (described as predominantly Caucasian) |
| Fisher  (2011b) | USA | Male & female sample (% unclear) | Unclear (described as predominantly Caucasian) |
| Fisher  (2013) | USA | 52% male, 48% female | Unclear (described as predominantly Caucasian) |
| Gmitrova (2013) | Slovak Republic | Male & female sample (% unclear) | Unclear |
| Goldstein (2018) | USA | 51% male, 50% female | 46% East Asian, 6% Central-/Latin-American, 5% Biracial, 4% Caribbean, 3% Black, 2% Southeast Asian, 1% Caucasian, 1% Arab, 32% Unclear |
| Golomb  (1977) | USA | Intervention: 47% male, 53% female  Control: 47% male, 53% female | Unclear |
| Han  (2010) | USA | Intervention: 50% male, 50% female  Control: 56% male, 44% female | Intervention: 67% Hispanic, 17% African American, 8% Biracial, 4% Caucasian, 4% Other  Control: 64% Hispanic, 32% African American, 4% Other |
| Jemutai  (2019) | South Africa, Kenya | Male & female sample (% unclear) | Unclear |
| Kalkusch (2020) | Switzerland | 50% male, 50% female | Unclear |
| Lau  (2005) | USA | 67% male, 33% female | 72% Caucasian, 11% African American, 8% Asian American, 6% Biracial, 3% Hispanic |
| Li  (2016) | China | 50% male, 50% female | Unclear |
| Morris  (2018) | Australia | Male & female sample (% unclear) | Unclear |
| O’Connor (2011) | Australia | Intervention: 58% male, 42% female  Control: 50 % male, 50% female | Unclear |
| Palma  (2014) | Portugal | 56% male, 44% female | Unclear |
| Pearson (2008) | USA | 56% male, 44% female | 77% Caucasian, 10% African American, 6% Other/No response, 4% Asian, 2% Latino/Hispanic |
| Pellegrini (1980) | USA | 50% male, 50% female | Unclear |
| Sawyer  (2019) | USA | Intervention: 53% male, 47% female  Control: 50% male, 50% female | Unclear (described as ‘racially and ethnically diverse’) |
| Schmitt  (2018) | USA | 44% male, 56% female | 78% Caucasian, 12% Multiracial/Other, 5% Hispanic, 3% Asian, 2% African American |
| Sinha  (2012) | USA | 52% male, 48% female | 50% Caucasian, 45% Black, 3% Native American, 2% Asian |
| Smith  (1978) | UK | 57% male, 43% female | Unclear |
| Smith  (1981) | UK | Intervention: 62% male, 38% female  Control: 39% male, 61% female | Unclear |
| Thibodeau (2016) | USA | Intervention: 49% male, 51% female  Control: 41% male, 59% female | Intervention: 95% Caucasian, 2.5% African American, 2.5% Other  Control: 85% Caucasian, 10% African American, 5% Other |
| Toub  (2018) | USA | 46% male, 54% female | 55% African American, 23% Hispanic/Latino, 14% Caucasian, 7% Other/Multiracial, 1% Asian |
| van schijndel (2010) | Netherlands | Intervention: 60% male, 40% female  Control: 58% male, 42% female | Unclear |
| *Note*. Information is provided for the whole sample of each study (intervention plus control) unless otherwise stated. | | | |
